# An enhanced Fracture Risk Evaluation Model (FREM) using national health data on morbidity and medications

**DOI:** 10.1093/jbmr/zjaf156

**Published:** 2025-10-31

**Authors:** Sören Möller, Marlene Rietz, Frederik Lykke Petersen, Jan Christian Brønd, Michael Kriegbaum Skjødt, Jens Søndergaard, Bo Abrahamsen, Katrine Hass Rubin

**Affiliations:** Research Unit OPEN, Department of Clinical Research, University of Southern Denmark, 5000 Odense, Denmark; Open Patient data Explorative Network (OPEN), Odense University Hospital, 5000 Odense, Denmark; Research Unit OPEN, Department of Clinical Research, University of Southern Denmark, 5000 Odense, Denmark; Division of Clinical Physiology, Department of Laboratory Medicine, Karolinska Institutet, 17177 Stockholm, Sweden; Open Patient data Explorative Network (OPEN), Odense University Hospital, 5000 Odense, Denmark; Department of Sports Science and Clinical Biomechanics, Research Unit for Exercise Epidemiology, Centre of Research in Childhood Health, University of Southern Denmark, 5230 Odense, Denmark; Research Unit OPEN, Department of Clinical Research, University of Southern Denmark, 5000 Odense, Denmark; Department of Medicine, Herlev Hospital, 2730 Copenhagen, Denmark; Research Unit of General Practice, Department of Public Health, University of Southern Denmark, 5230 Odense, Denmark; Research Unit OPEN, Department of Clinical Research, University of Southern Denmark, 5000 Odense, Denmark; Department of Medicine 1, Holbæk Hospital, 4300 Holbæk, Denmark; Research Unit OPEN, Department of Clinical Research, University of Southern Denmark, 5000 Odense, Denmark; Open Patient data Explorative Network (OPEN), Odense University Hospital, 5000 Odense, Denmark

**Keywords:** major osteoporotic fracture, hip fracture, automated risk calculation, decision support tool, risk prediction, screening, register data, administrative health data, general practice, osteoporosis

## Abstract

Osteoporosis is a major health concern in older individuals. Efficient case-finding is essential for timely risk assessment and treatment for high-risk patients. To prevent fractures and reduce the risk of subsequent disability, approaches offering clinically sufficient sensitivity with acceptable specificity are warranted. Although pharmaceutical osteoporosis treatment is effective, it is often diagnosed at a late stage, for instance, following a fracture. The aim of this study was to extend the existing Fracture Risk Evaluation Model (FREM), which identifies individuals at risk of an imminent (1-yr) major osteoporotic fracture (MOF) based on administrative health data. This extension (FREM_Ver2_) included data on morbidity and medications and evaluated age-specific risk cut-offs to enhance the risk assessment of MOF and hip fracture (HF) risk, respectively. We included the entire population of Denmark aged ≥45 yr at baseline (2022; *N* = 2 493 180), not previously diagnosed with osteoporosis or receiving osteoporosis treatment. The cohort was divided into 4 groups stratified by sex and age (<65 yr, ≥65 yr). Each of the 4 groups was randomly split into a 60% development, a 20% model validation, and a 20% cut-off validation cohort. All diagnoses from Danish hospitals and filled prescriptions from Danish pharmacies from 2007 to 2021 were used as possible predictors for MOF. These predictors correspond to information that in Denmark is automatically transferred to general practitioner’s electronic health records; hence, prediction would be possible in general practice. Models were constructed by logistic regression with LASSO regularization, determining the preferred regularization hyper parameter by cross-validation and forcing categorical age to be included. Across subgroups, the models obtained poor to acceptable area under the curves (AUCs) of 0.656-0.714 for MOF, and acceptable AUCs of 0.728-0.764 for HFs. Additionally, the models achieved sensitivities of around 80% or higher in almost all subgroups. This performance, together with the available predictors, makes FREM_ver2_ a feasible decision support system as a step toward an opportunistic screening program in health care settings with access to administrative data.

## Graphical Abstract

**Graphical Abstract:**
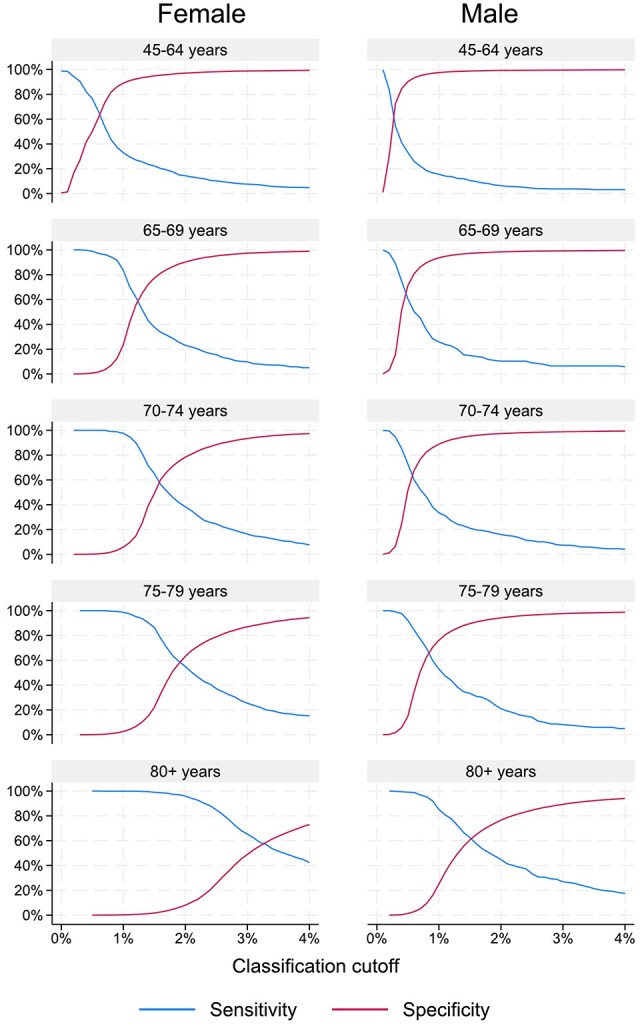

## Introduction

Osteoporosis is a common skeletal disease, which poses a growing public health challenge due to increased bone fragility and fracture risk.^1^^,^^2^ Despite its widespread prevalence, the condition is underdiagnosed and undertreated, leaving many individuals vulnerable to fractures without timely care.^3–6^ To address this, improved case-finding and fracture risk assessment strategies are crucial. Various approaches have been proposed,^7–9^ including clinical decision support systems and machine learning algorithms that can automatically alert clinicians to individuals at high risk of osteoporotic fractures, potentially enabling automated, opportunistic screening without the need for manual data entry.

We developed the Fracture Risk Evaluation Model (FREM) to identify individuals over the age of 45 yr who are at high risk of experiencing an osteoporotic fracture within 1 yr.^10^ It utilizes routinely collected diagnosis data from the national Danish registers, and the predictors were selected unsupervised, that is, based on their statistical performance rather than physiology or causality.^10^ FREM_ver1_ has been validated internally and externally using an updated extraction of Danish register data and Canadian health data.^11^^,^^12^ The algorithm is intended for the integration into electronic health records at the general practitioner (GP), where it can provide automated easily interpretable risk estimates without requiring manual data entry. Consequently, it has been suggested as an opportunistic screening tool for primary healthcare providers to identify patients that should be referred to a DXA scan.^7^^,^^8^ This can make FREM a valuable data-driven decision-support tool, enhancing the efficiency of case-finding and enabling timely intervention strategies for patients. However, the approach is currently limited by the low sensitivity of FREM_ver1_, which is challenging as an opportunistic screening tool is required to achieve a high sensitivity_._ Increasing the accuracy of FREM might be possible by using more advanced statistical methods or adding additional information, such as medication data. We are presently evaluating the usability of implementing the algorithm in general practice.

The aim of this study is to investigate an enhanced version of FREM (FREM_ver2_), integrating additional register data and evaluating age-specific risk cut-offs to improve the risk assessment of major osteoporotic fractures (MOFs) and hip fractures (HFs), respectively. As will be discussed below, the weighing of sensitivity vs specificity is essentially a decision related to clinical use and health economics, but we prioritized a high sensitivity. Thereby, the goal is to avoid providing false reassurance to patients while bearing in mind that the referral to DXA is a safe procedure, albeit in many countries a scarce resource that needs to be appropriately targeted.

## Materials and methods

### Study design and study population

This study was conducted as a nationwide register-based cohort study based on a data from the Danish national registers covering all citizens alive and living in Denmark on January first, 2022 (index date) aged 45 yr or above. The data extraction included 15 yr of retrospective data (2007-2021) and 1 yr of follow-up data (2022).

*Exclusion criteria*: We excluded all individuals who—in the 15-yr look-back period—had a filled prescription for an osteoporosis medication and/or a diagnosis code for hospital administration of a bisphosphonate or denosumab, as well as individuals with a diagnosis code for osteoporosis in the National Patient Register (Table S1). A prior fracture on its own did not result in exclusion.

*Surveillance limitations:* Individuals who died or emigrated during the index year (2022), as well as individuals with incomplete look-back period were included in the model. For these individuals, the model assumed there had been no occurrence of predictors and outcomes in the unobserved periods.

### Data sources

#### The Danish national registers

Data sources consisted of three Danish national registers: The Civil Registration System (CRS), the Danish National Patient Register (NPR), and the Danish National Prescription Register (DNPR). The Danish national registers cover the entire population of Denmark and offer long-term high quality data, which makes it possible to conduct population-based studies.^13^

The CRS registers all individuals alive and living in Denmark for administrative purposes, including the unique personal identification number. This number was used for linkage to the other registries.^13^ The CRS was used to identify individuals for inclusion in the study population and to determine age, sex, death, and emigration.^14^^,^^15^

The NPR contains information on all inpatient, outpatient, and emergency department contacts from the Danish hospitals.^16^ The records contains specific information on primary and optional secondary diagnoses registered according to the International Classification of Diseases (ICD) and on performed procedures and operations.^16^ The NPR was used to define outcomes and identify diagnoses as predictors.

The DNPR contains information on filled prescriptions of medicines sold in Danish pharmacies. The register provides information on ATC-codes and redemption dates. The DNPR was used to identify medications as predictors.^17^

### Outcomes (fractures)

The primary outcome was MOF. Hip fracture is included as a secondary outcome, as HFs are known to be strongly associated with frailty and short-term mortality, making them an important outcome on their own. Outcomes were defined as suggested and validated in a study by Clausen et al.^18^ In short, an incident HF is defined by a relevant diagnosis code (ICD10: S720, S721, or S722) followed by a relevant surgical procedures code (NFB* or NFJ4-9) within 7 d of hospitalization. A MOF is defined as either a HF or a fracture in the humerus, the clinical vertebral area or distal forearm, followed by a relevant imaging procedure code within 7 d of hospitalization.^18^

For either fracture outcome (MOF or HF), only the first incident fracture in 2022 was included as an outcome, additionally requiring a grace period of 90 d from earlier fracture (ie, occurring in late 2021), to avoid counting follow-up hospital visits for prior fractures as an incident fracture.

### Predictors: conditions of interest

Predictors used in the risk prediction of incident MOF and HF:

- Age on January first, 2022, stratified into 5-yr age groups, with an open-ended group 80+ yr of age, due to low numbers of individuals with ages above 85 yr.
- Hospital diagnoses at ICD-10 level 3 (eg, M80) with a prevalence of at least 0.1% within the development cohorts. Hospital diagnoses were included as dichotomous predictors (yes/no).
- Filled prescriptions at ATC second level (eg, N03) with a prevalence of at least 0.1% within the development cohorts as a dichotomous variable (yes/no).

Medication and diagnosis data used to identify predictors was collected within the look-back period of 15 yr, and these data would be available in the electronic health record at the GP.

#### Other measures (comorbidity)

The Charlson Comorbidity Index (CCI) was generated from the NPR data during the period from 2007 to 2021 at the index date to assess the burden of comorbidity among the respondents.^19^

### Statistical analysis

Based on our previous findings implicating age and sex as a key predictor of fracture risk,^10^ we stratified the cohort into 4 groups using sex and age, classifying individuals aged 45-64 yr and 65+ yr. We randomly assigned individuals from each of the four groups into a 60% development, a 20% model validation (to be used for determining included predictors), and a 20% cut-off validation cohort (to be used to determine a relevant risk cut-off for referral to DXA-scan). For reproducibility of the results, a random seed was set.

#### Descriptive statistics

Descriptive statistics are reported stratified by sex and age (45-64 yr and 65+ yr, respectively). Median and IQR are reported for age. Categorical variables are reported as counts and proportions.

#### Model development

A total of 8 prediction models were developed, 1 for each combination of sex and age group and with both MOF and HF outcomes. Each prediction model was constructed using logistic regression with LASSO regularization determining hyper parameters by 10-fold cross-validation and forcing categorical age (5 yr increments) to be included in the model, as age is a strong predictor of MOF and HF. See our published protocol paper for details.^20^

#### Model validation

The validity of the final models was investigated using the model validation cohort separately for each outcome and group. Area under the curves (AUCs) (with 95% CI) were determined from ROC curves, and compared to AUCs obtained from a model including only age, as well as age and diagnoses (similar to the original FREM_ver1_). Area under the curves were classified as poor (0.5-0.7), acceptable (0.7-0.8), excellent (0.8-0.9), or outstanding (>0.9) using the nomenclature suggested by Hosmer and Lemeshow.^21^ Furthermore, sensitivity, specificity, PPV, and NPV for cut-offs corresponding to 0.1%-4% 1-yr risk of fractures were estimated. From these estimates, we determined the highest cut-off that preserved a sensitivity of approximately 80%, and separately, a sensitivity of approximately 90%, for each unique stratified model. This was conducted stratified due to the large variation in fracture incidence across age and sex. The selected cut-off was subsequently evaluated in the cut-off validation cohort reporting sensitivity, specificity, PPV and NPV, and AUCs.

Moreover, we evaluated the performance of FREM_ver2_ for 2-yr fracture risk prediction. Lastly, we evaluated the performance for both 1- and 2-yr fracture risk prediction using 5- and 15-yr lookback periods, respectively. The validation steps were repeated for these scenarios. For the 2-yr risk prediction, the determined 1-yr cut-offs were doubled, which was done in alignment with suggestions from Leslie et al.^22^

All analyses were performed with Stata statistical software (version 18; StataCorp).

#### Sensitivity analyses

Model development used 10-fold cross-validation to facilitate predictive performance. To investigate the robustness of these models, a sensitivity analysis was conducted in which the optimal hyper parameter for LASSO was determined by Bayes information criterion (BIC). Moreover, we repeated the main analysis with LASSO using fourth level ATC codes (eg, A10AB instead of A10). Lastly, backwards selection instead of LASSO was assessed in the main analysis.

## Results

**Figure 1:**
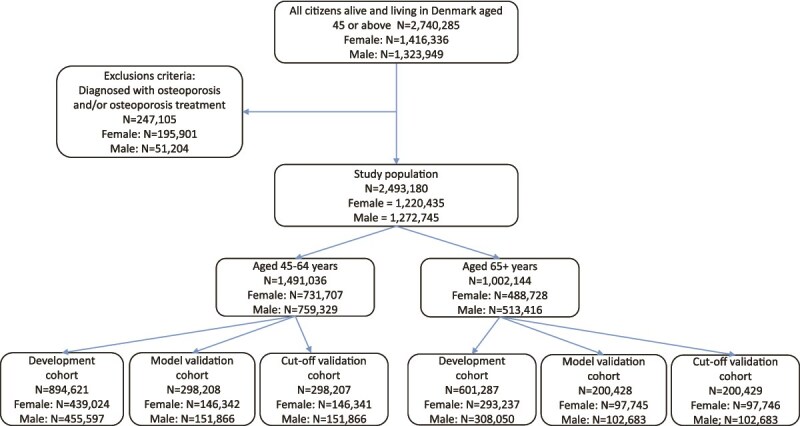
Flowchart participants.

As of January 1, 2022, Denmark had a total population of 2 740 285 individuals aged 45 yr or older. After excluding the 9.0% already diagnosed with osteoporosis or receiving osteoporosis treatment (*N* = 247 105, 79% of whom were females), 2 493 180 individuals remained eligible for the study. Among these, 731 707 females were aged 45-64 yr and 488 728 were 65+ yr. The corresponding numbers for males were 759 329 and 513 416, respectively (Figure 1).

Population characteristics are shown in Table 1. The median age for both females and males in the younger (45-64 yr) cohort was 55 yr (IQR 50; 59). In the 65+ age cohorts, the median age for both sexes was 74 yr, with IQR of 69-80 yr for females, and 69-79 yr for males.

**Table 1:** Study population characteristics.

	**Total**	**Females**		**Males**	
		**45-64 yr**	**65+ years**	**45-64 yr**	**65+ yr**
	***N* = 2 493 180** **(100%)**	***N* = 731 707** **(29.3%)**	***N* = 488 728** **(19.6%)**	***N* = 759 329** **(30.5%)**	***N* = 513 416** **(20.6%)**
**Age, years (median, (Q1; Q3))**	61 (53; 72)	55 (50; 59)	74 (69; 80)	55 (50; 59)	74 (69; 79)
**Age categorized, *N* (%)**					
**45-49**	383 801 (15.4)	191 576 (26.2)	-	192 225 (25.3)	-
**50-54**	383 442 (15.4)	189 101 (25.8)	-	194 341 (25.6)	-
**55-59**	396 559 (15.9)	193 149 (26.4)	-	203 410 (26.8)	-
**60-64**	327 234 (13.1)	157 881 (21.6)	-	169 353 (22.3)	-
**65-69**	289 610 (11.6)	-	138 472 (28.3)	-	151 138 (29.4)
**70-74**	264 696 (10.6)	-	126 339 (25.9)	-	138 357 (26.9)
**75-79**	227 210 (9.1)	-	108 341 (22.2)	-	118 869 (23.2)
**80.0+**	220 628 (8.8)	-	115 576 (23.6)	-	105 052 (20.6)
**Incident MOF in 2022, *N* (%)**	22 308 (0.9)	4611 (0.6)	10 573 (2.2)	2452 (0.3)	4672 (0.9)
**45-49**	860 (0.2)	405 (0.2)	-	455 (0.2)	-
**50-54**	1464 (0.4)	907 (0.5)	-	557 (0.3)	-
**55-59**	2333 (0.6)	1603 (0.8)	-	730 (0.4)	-
**60-64**	2406 (0.7)	1696 (1.1)	-	710 (0.4)	-
**65-69**	2667 (0.9)	-	1879 (1,4)	-	788 (0.5)
**70-74**	3103 (1.1)	-	2181 (1.7)	-	922 (0.7)
**75-79**	3378 (1.5)	-	2278 (2.1)	-	1100 (0.9)
**80.0+**	6097 (2.8)	-	4235 (3.7)	-	1862 (1.8)
**Incident HF in 2022, *N* (%)**	5918 (0.2)	294 (0.0)	3199 (0.7)	348 (0.0)	2077 (0.4)
**Incident MOF in 2022/2023 (2 yr), *N* (%)**	28 038 (1.1)	5850 (0.8)	13 209 (2.7)	3123 (0.4)	5856 (1.1)
**Incident HF in 2022/2023 (2 yr), *N* (%)**	7495 (0.3)	359 (0)	4017 (0.8)	454 (0.1)	2665 (0.5)
**Previous MOF before 2022 (5 yr), *N* (%)**	62 250 (2.5)	13 892 (1.9)	28 925 (5.9)	9036 (1.2)	10 397 (2.0)
**Previous HF before 2022 (5 yr), *N* (%)**	9093 (0.4)	531 (0.1)	4713 (1.0)	914 (0.1)	2935 (0.6)
**Previous MOF before 2022 (15 yr), *N* (%)**	132 686 (5.3)	27 095 (3.7)	61 607 (12.6)	21 788 (2.9)	22 196 (4.3)
**Previous HF before 2022 (15 yr), *N* (%)**	15 630 (0.6)	841 (0.1)	7963 (1.6)	1780 (0.2)	5046 (1.0)
**Charlson comorbidity index, grouped**					
**0**	2 000 859 (80.3)	632 529 (86.4)	354 830 (72.6)	669 307 (88.1)	344 193 (67.0)
**1-2**	402 586 (16.1)	87 791 (12.0)	107 489 (22.0)	77 417 (10.2)	129 889 (25.3)
**3+**	89 735 (3.6)	11 387 (1.6)	26 409 (5.4)	12 605 (1.7)	39 334 (7.7)
**Death 2022**	43 037 (1.7)	2010 (0.3)	16 122 (3.3)	3701 (0.5)	21 204 (4.1)
**Emigration 2022**	4344 (0.2)	1281 (0.2)	221 (0.0)	2540 (0.3)	302 (0.1)
**Incomplete lookback (2007-2021)**	55 040 (2.2)	18 740 (2.6)	3479 (0.7)	27 016 (3.6)	5805 (1.1)

Abbreviations: HF, hip fracture; MOF, major osteoporotic fracture.

A total of 43 037 (1.7%) of individuals died during follow-up (2022), while 4344 (0.2%) emigrated, and 55 040 (2.2%) had an incomplete look-back period (Table 1).

No differences between the development, model validation, and cut-off validation cohorts were observed in any of the 4 groups, indicating the validity of the random assignment (Table S2).

### Fracture outcomes

In 2022, 0.6% (*N* = 4611) of females and 0.3% (*N* = 2452) of males aged 45-64 yr experienced a MOF. In the older age group (65+), the number of MOF events was 2.2% (*N* = 10 573) and 0.9% (*N* = 4672) for females and males, respectively (Table 1). Less than 0.1% of both females (*N* = 294) and males (*N* = 348) in the age group 45-64 yr experienced a HF during 2022. In the age group 65+, the HF incidence was 0.7% (*N* = 3199) for females and 0.4% (*N* = 2077) for males (Table 1).

### Conditions (diagnoses and medications) related to fractures and development of the predictive model (FREMver2)

#### Age group 45-64 yr

In the age group 45-64 yr, we identified 1666 and 1578 distinct ICD-10 codes for females and males, respectively. After excluding administrative DZ codes and ICD-10 codes with a prevalence below 0.1%, 623 potential ICD-10 codes for females and 534 for males remained for inclusion in the model selection procedures. A total of 86 ATC codes were identified for both females and males. After excluding ATC codes with a prevalence below 0.1%, 70 and 67 potential ATC remained for inclusion in the model selection in females and males, respectively (Figure S1).

For prediction of MOF, the LASSO regression selected 80 and 70 of these ICD-10 codes and 12 and 10 ATC codes, for females and males, respectively (Table S3). The strongest predicting diagnoses in females were previous fractures (S42 and S52) and symptoms and signs involving emotional state (R45) in contrast to herpes zoster (B02) and fracture of cervical vertebra and other parts of neck (S12) as well as fractures of shoulder and upper arm (S42) in males. The strongest predicting medications were pancreatic hormones (H04) in both sexes, other nervous system drugs (N07), and digestives (A09) in females, and drugs for constipation (A06) and antiepileptics (D02) in males. Similarly, for prediction of HF—an extremely rare outcome in this age group—the LASSO regression selected 37 and 60 ICD-10 codes and 7 and 8 ATC codes, for females and males, respectively (Table S4).

#### Age group 65+

In the age group 65+ yr, we identified 1588 and 1545 distinct ICD-10 codes for females and males, respectively. After excluding administrative DZ codes and ICD-10 codes with a prevalence below 0.1%, 577 potential ICD-10 codes for females and 593 for males remained for inclusion in the model selection procedures. A total of 85 and 84 ATC codes were identified for females and males, respectively. After excluding ATC codes with a prevalence below 0.1%, 71 potential ATC codes for females and 69 for males remained for inclusion in the model selection (Figure S1).

The LASSO regression selected 115 and 116 of these ICD-10 codes and 16 and 20 ATC codes, for females and males, respectively (Table S3). The strongest predicting diagnoses were previous fractures of shoulder and upper arm (S42) and blindness and low vision (H54) for females and HIV disease resulting in other conditions (B23) and multiple myeloma and malignant plasma cell neoplasms (C90) for males. The most powerful medication features were bile and liver therapy (A05), pancreatic hormones (H04), and digestives, including enzymes (A09) for females and gynecological anti-infectives and antiseptics (G01) (these medications, especially clindamycin, are also to a limited degree prescribed to males in Denmark), and digestives, including enzymes (A09) for males. Similarly, for prediction of HF, the LASSO regression selected 146 and 117 ICD-10 codes and 25 and 17 ATC codes, for females and males, respectively (Table S4).

### Validation of FREM_ver2_

We estimated an AUC for MOF prediction of 0.702 (0.684; 0.719) for females and 0.672 (0.645; 0.699) for males aged 45-64 yr in the model validation cohorts. In individuals 65+ yr, we computed AUCs of 0.656 (0.644; 0.668) for females and 0.714 (0.696; 0.731) for males.

**Figure 2:**
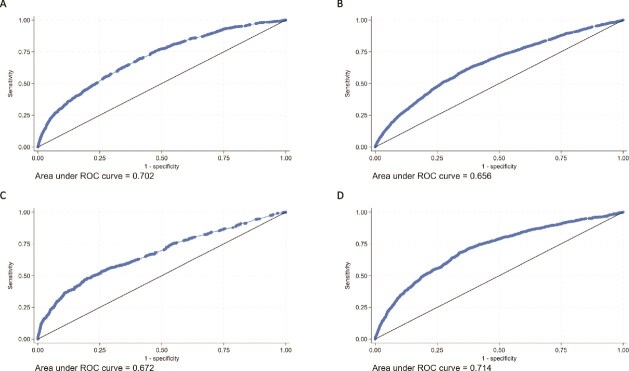
Predictive performance of FREM_ver2_ for predicting 1-yr MOF risk as evaluated by receiver operating characteristics (ROC) curves and area under the curve (AUC). (A) Females aged 45-64 yr, (B) females aged 65+ yr, (C) males aged 45-64 yr, and (D) males aged 65+ yr.

**Figure 3:**
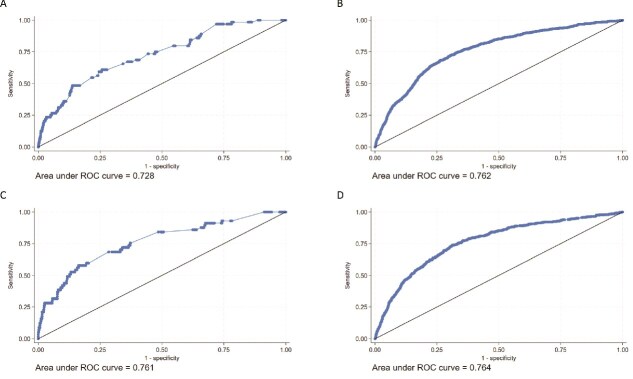
Predictive performance of FREM_ver2_ for predicting 1-yr HF risk as evaluated by receiver operating characteristics (ROC) curves and area under the curve (AUC). (A) Females aged 45-64 yr, (B) females aged 65+ yr, (C) males aged 45-64 yr, and (D) males aged 65+ yr.

**Figure 4:**
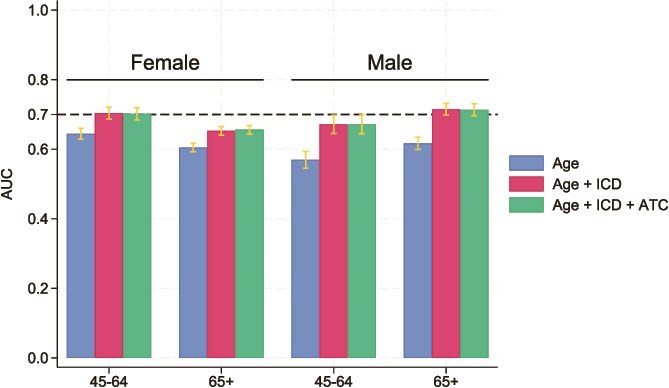
Comparison of area under the curve (AUC) for predicting MOF between models including age only, age and diagnoses and FREM_ver2_.

For HF, an AUC of 0.728 (0.665; 0.790) for females and 0.761 (0.693; 0.828) for males was determined in the age group 45-64 yr. Finally, in the age group 65+ yr, we estimated AUCs of 0.762 (0.743; 0.781) for females and 0.764 (0.740; 0.787) for males (Table 2, Figure 2 (MOF) and Figure 3 (HF)). Over all 4 subgroups (sex and age), the AUCs obtained for FREM_ver2_ were significantly different (non-overlapping CI’s) when adding more predictors than just age for predicting MOF (Figure 4), whereas for predicting HF (Figure S2), only males 65+ yr old had significantly higher AUCs when adding more predictors than just age. However, in all cases, the inclusions of medications did not significantly improve predictions over including just age and diagnoses.

**Table 2:** Predictive performance of FREM_ver2_ and sensitivity and specificity of suggested risk cut-offs.

**Sex, age groups, years**	**Development cohort AUC (95% CI)**	**Model validation cohort AUC (95% CI)**	**Cut-off validation cohort AUC (95% CI)**	**Original FREM AUC (95% CI)** ^**a**^	**Age groups, years**	**Suggested cut-off**	**Sensitivity (in the cut-off validation cohort)**	**Specificity (in the cut-off validation cohort)**
**Major osteoporotic fracture**								
**Females**								
**45-64**	0.726 (0.716; 0.735)	0.702 (0.684; 0.719)	0.693 (0.677; 0.710)	0.704 (0.688; 0.720)	45-64	0.4%	81%	41%
**65+**	0.681 (0.674; 0.688)	0.656 (0.644; 0.668)	0.650 (0.637; 0.662)	0.659 (0.647; 0.671)	65-69	1.0%	84%	24%
					70-74	1.3%	80%	25%
					75-79	1.6%	79%	33%
					80+	2.5%	82%	26%
**Males**								
**45-64**	0.701 (0.686; 0.716)	0.672 (0.645; 0.699)	0.657 (0.630; 0.683)	0.656 (0.629; 0.682)	45-64	0.2%	84%	31%
**65+**	0.737 (0.727; 0.746)	0.714 (0.696; 0.731)	0.718 (0.701; 0.735)	0.714 (0.698; 0.730)	65-69	0.4%	79%	51%
					70-74	0.5%	76%	51%
					75-79	0.6%	81%	34%
					80+	1.1%	81%	33%
**Hip fracture**								
**Females**								
**45-64**	0.867 (0.840; 0.894)	0.728 (0.665; 0.790)	0.764 (0.704; 0.823)	0.771 (0.717; 0.825)	45-64	0.1%	31%	95%
**65+**	0.805 (0.796; 0.815)	0.762 (0.743; 0.781)	0.747 (0.727; 0.767)	0.762 (0.743; 0.780)	65-69	0.1%	84%	23%
					70-74	0.2%	80%	27%
					75-79	0.4%	76%	40%
					80+	1.1%	79%	38%
**Males**								
**45-64**	0.825 (0.793; 0.857)	0.761 (0.693; 0.828)	0.761 (0.692; 0.830)	0.677 (0.607; 0.748)	45-64	0.1%	36%	94%
**65+**	0.810 (0.798; 0.822)	0.764 (0.740; 0.787)	0.766 (0.743; 0.790)	0.762 (0.740; 0.784)	65-69	0.1%	88%	50%
					70-74	0.1%	87%	19%
					75-79	0.3%	77%	55%
					80+	0.6%	76%	42%

a In the model validation cohort.

The AUCs on the scenario with 5-yr lookback and 1-yr risk prediction were similar to those observed in the main analyses. We estimated an AUC for MOF prediction of 0.707 (0.690; 0.724) for females and 0.672 (0.645; 0.699) for males aged 45-64 yr, and AUCs of 0.647 (0.635; 0.660) for females and 0.734 (0.718; 0.751) for males aged 65+ yr. For HF, an AUC of 0.814 (0.753; 0.875) for females, and 0.757 (0.690; 0.824) for males in the age group 45-64 yr and 0.761 (0.742; 0.780) for females, and 0.795 (0.774; 0.816) for males aged 65+ were determined.

In the scenarios with a 2-yr risk prediction window, among females aged 45-64 yr, we obtained AUCs for MOF prediction of 0.691 (0.679-0.704) with a 15-yr lookback and 0.680 (0.668-0.693) with a 5-yr lookback. Among males aged 45-64 yr, the corresponding AUCs were 0.674 (0.655-0.693) and 0.651 (0.631-0.670). For females aged 65 yr and older, AUCs were 0.645 (0.636-0.654) and 0.636 (0.627-0.645), and for males aged 65 yr and older, 0.721 (0.710-0.733) and 0.714 (0.702-0.726) for the 15- and 5-yr lookback periods, respectively.

The corresponding results for HF with a 2-yr risk prediction window among females aged 45-64 yr were 0.794 (0.754-0.835) for the 15-yr lookback and 0.778 (0.738-0.818) for the 5-yr lookback. Among males aged 45-64 yr, the AUCs were 0.771 (0.728-0.814) and 0.753 (0.713-0.793) for the 15- and 5-yr lookback periods, respectively. For females aged 65 yr and older, we obtained 0.755 (0.741-0.768) and 0.753 (0.740-0.766), and for males aged 65 yr and older, 0.769 (0.754-0.784) and 0.763 (0.748-0.778) for the 15- and 5-yr lookback periods, respectively (Table S6).

### Determination of risk cut-offs for FREM_ver2_

**Figure 5:**
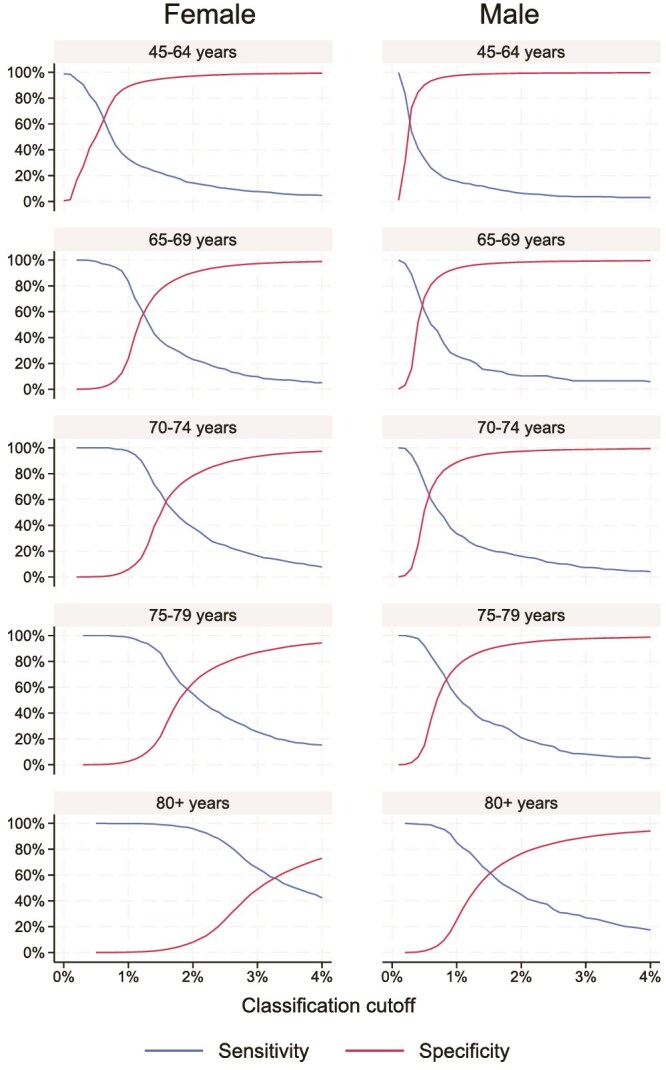
Sensitivity and specificity for prediction of 1-yr MOF risk evaluating cut-offs from 0.1% to 4%.

**Figure 6:**
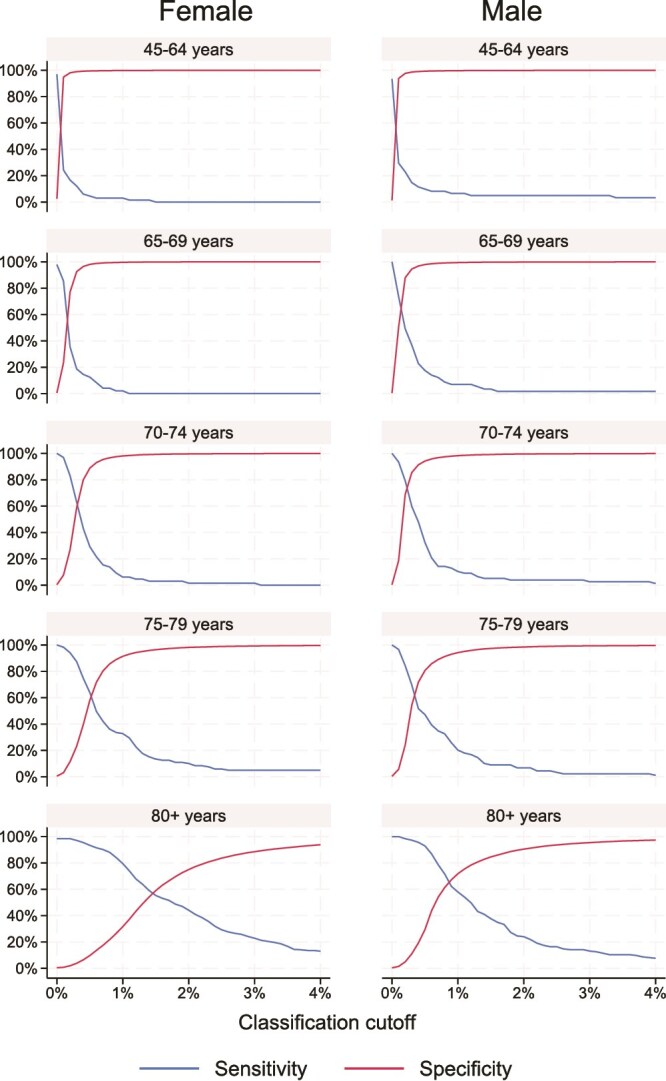
Sensitivity and specificity for prediction of 1-yr HF risk evaluating cut-offs from 0.1% to 4%.

The estimated sensitivity and specificity (Figures 5 and 6) and PPV and NPV (Figures S3 and S4) were determined separately for each 5-yr age group above age 65 and for the whole group below age 65 to account for the limited number of fractures in the younger group. Based on these results, we selected a cut-off for each age group and sex. The identified cut-offs for 80% sensitivity range between 0.2% and 2.5% for MOF and between 0.1% and 1.1% for HF (Table 2). Moreover, for 90% sensitivity, we selected similar but slightly lower cut-offs (Table S5). We then applied the cut-offs identified in the model validation cohort on the cut-off validation cohort, achieving sensitivities similar to those aimed for and specificities of at least 24% in all age groups for MOF. The performance was similar for HF in those aged 65+ yr, whereas only a sensitivity of 31% (females), respectively, 36% (males) could be achieved for HF for the age group 45-65 (Table 2).

Sensitivities and specificities obtained for the scenarios with 5-yr lookback as well as with 2-yr risk prediction (with both 5- and 15-yr lookback) were comparable to the main analyses (Table S7).

### Sensitivity analyses

Optimizing the LASSO based on BIC instead of cross-validation, resulted in inclusion of too few predictors for meaningful validation (data not shown). Moreover, the LASSO analysis using ATC codes on fourth level (eg, A10AB) instead of second level (eg, A10), and the analysis using backwards selection instead of LASSO, both resulted in similar results as the main analysis, and we did not proceed with the formal validation of these models (data not shown).

## Discussion

### Main findings

This study builds upon the original FREM_Ver1_, which utilizes diagnosis data obtained from health registries.^10^ The key objective is to provide a case-finding algorithm for identifying individuals at high 1-yr risk of fracture who are not already known to have osteoporosis or receiving anti-osteoporosis medications. Importantly, diagnoses included in the model came from hospital contacts only, likely identifying more severe cases of the respective diseases. The added medication data, however, also included primary care prescriptions, capturing not only severe but also mild comorbidities. Here, we extend the model by incorporating medications, stratifying the tool into separate models for the age groups 45-64 yr and 65+ yr, and refine the statistical methodology. These changes generally enhanced the predictive performance of the model compared to the original FREM_ver1_, and the distinct cut-offs by age and sex enable design of tailored screening strategies. This may help to develop a care path in a model that might be feasible for implementation in automated opportunistic screening.

### Comparison with other studies

#### Comparison to the original FREM_ver1_

The original FREM_ver1_ included 38 and 43 hospital diagnoses as predictors for MOF in females and males, respectively, with an accuracy resulting in an AUC of 0.750 for females and 0.752 for males.^10^ The extended FREM_ver2_ suggested in this paper in general obtained similar AUCs (0.656-0.714) for MOF, although it included a larger number (80-136) of predictors, still with age as the dominating factor explaining fracture risk. Similarly, while the FREM_ver1_ included 32 predictors for HF for each sex, obtaining AUCs of 0.874 in females and 0.851 in males, this paper’s algorithms include 44-171 in the HF prediction models, achieving AUCs from 0.728 to 0.762. As expected, the driving risk predictors were age, as well as previous fractures, consistent with both FREM_ver1_ and other existing prediction tools, including FRAX.

The inclusion of additional predictors, including medications, and the more advanced statistical approach, did not markedly improve the AUC neither for MOF nor HF prediction. However, some medications were included in the prediction models as strong individual predictors for fractures. This apparent contradiction can be partly explained by the fact that LASSO regression does not optimize directly toward a maximized AUC, but rather tries to minimize the deviation between observed and predicted outcomes for each individual in the dataset.

On the other hand, the age-specific models and risk cut-offs suggested in this paper, resulted in a higher sensitivity than FREM_ver1_, which would be necessary for clinical implementation in opportunistic screening. While the additional data—to our surprise—did not improve the prediction, the more detailed handling of age resulted in better predictive performance, especially for the older age groups. This might be due to the additional predictors, instead of providing further predictive power, diluting the available information, resulting in less parsimonious models.

#### Comparison to other existing fracture prediction models

Compared to the highly used FRAX algorithm,^23^ FREM_ver2_ does not include predictors that require manual data input—a factor which may introduce errors and require additional efforts from the healthcare professional. Therefore, FREM_ver2_ allows for feasible and automatic application based on electronic health records, for instance, the medical journal system used by GPs in Denmark. Other predictive tools, using only administrative data, have been suggested, including the deep-learning algorithm crystal bone.^24^ Briefly, crystal bone estimates a 1- to 2-yr fracture risk using electronic health records and machine learning techniques commonly employed in natural language processing with an AUC of 0.81. However, the algorithm is proprietary and thus costly for health systems to utilize. Moreover, even though the Crystal Bone method has been described in detail, the replication of the model in a different context may be limited if diagnoses are missing, not registered correctly, or registered differently across countries. Similarly, the FRAX algorithm is proprietary, and details of the algorithm are not publicly available.^25^ Implementation of risk prediction models in clinical care would require sharing the model structure publicly in accordance with High Risk AI System classifications discussed in the Artificial Intelligence Act of the European Union,^26^ as well as supporting the interpretation of model outputs using explainable AI methodology.^27^

### Implementation considerations

We propose that FREM_ver2_ could serve as a decision-support tool in in those practices or hospital settings with access to administrative data, acting as a first step for osteoporosis case-finding in an opportunistic screening program, although only for those aged 65 or older. This type of an open-source tool has been requested by scientific societies as a necessary step to improve osteoporosis management.^8^^,^^9^ Similarly, initiatives are underway in Italy with the Osteoporosis Diagnostic and Therapeutic Pathway strategy.^28^

FREM_ver2_ aligns with the “opportunistic screening” approach in the fracture risk prediction framework recently proposed by Hong et al.^7^ This approach is expected to counteract the adverse selection seen in many screening programs, where high risk individuals often show lower participation rates,^29^ thereby impacting the effectiveness of the program.^30^ For example, in the ROSE screening trial,^31^ participants completed a questionnaire that allowed for FRAX-based calculation of the 10-yr fracture risk. However, individuals at high fracture risk were less inclined to participate than those of low risk.

Since all FREM_ver2_ predictors are available in the electronic health record, the system could automatically alert the GP of patients at a high risk of imminent fractures, prompting them to invite these patients for consultations. During these consultations, fracture risk could be further assessed, for instance taking into account lifestyle factors. If necessary, patients could be referred for a DXA scan. Such an implementation would require a decision on a suitable cut-off, preferably stratified by age and sex. In this paper, we report possible cut-offs for obtaining 80%, respectively, 90% sensitivity, although a suitable cut-off for implementation would depend on resource considerations, balancing the amount of DXA scans performed with the number of avoided fractures.^32^ As the suggested application of FREM_ver2_ is in opportunistic screening, referring high-risk patients to DXA scanning, the cut-offs favor a high sensitivity as long as economically feasible.^32^ Determining a suitable level for risk cut-offs that is both acceptable and useful to patients and clinicians is challenging and requires further study. Our currently proposed cut-offs may be considered low for initiating treatment, but we regard them as suitable as a first step in opportunistic screening. Moreover, they align well with cut-offs suggested for an age-dependent intervention threshold suggested in a publication by Kanis et al.^33^ Here, one should consider the important distinction between targeting DXA and targeting pharmaceutical treatment. Treating osteoporosis involves a lengthy treatment with some risk of serious adverse events, whereas DXA referral involves 1 clinic visit to undergo a safe, low radiation examination. Hence, DXA scans do not put low-risk individuals falsely classified to high-risk screening at risk of resulting complications. By contrast, if the case finding path leads directly to pharmaceutical intervention, as is the case for FRAX in some but not all countries, then a higher specificity will be required to justify implementation. With our currently proposed cut-offs, the performance of FREM_ver2_ is limited in the younger age group (45-64 yr) obtaining only poor performance according to AUC for MOF, especially if 1 desires a sensitivity of 90%. For HFs, AUCs were acceptable in these younger age groups, but sensitivity was low when using a 0.1% cut-off. The cut-offs proposed for individuals at the age of 65+ yr are considered of sufficient performance to be useful for opportunistic screening. As osteoporotic fractures mainly occur in individuals aged 65+ yr, this would be realistic, although future improvements of the model for younger age groups is desirable. Moreover, FREM_ver2_ has only been evaluated in a Danish setting which limits generalization to other datasets and populations prior to external validation.

### Strengths and limitations

This study has some limitations. While the outcomes have been validated, specific predictors from the registries have not all been explicitly validated. Furthermore, as in all such studies, there is a risk of erroneous (coding errors) or inconsistent use of ICD diagnoses. It has, however, been shown that diagnoses and medication registrations in Danish health registries generally have high validity.^16^^,^^17^ Especially, as vertebral fractures are often not clinically diagnosed, the capture of these fractures is likely incomplete in this—as well as in other—registry-based research projects. Further, many patients with severe osteoporosis will not suffer a MOF in a time scale of 1 yr, so the imminent risk scenario may limit sensitivity. For example, in UK guidance,^34^ at age 70 the 10-yr MOF risk marking *very high risk* is 30%, so in each year 97% of this group will not suffer a MOF. An additional limitation is that diagnoses given in general practice, over-the-counter medication, and data on lifestyle factors are not available in the registers, which can imply that access to other strong predictors of fracture risk could have improved our algorithm; in the planned implementation in general practice, primary practice diagnoses will be available for inclusion. Next, it is important to appreciate that the included predictors only indicate association with fractures with no proof of causality. For a case-finding algorithm, specifically, this is not an issue so long as the predictors captured are reliable proxies for risk factors in the population of interest and do not modify the effect of a subsequent anti fracture intervention with medications. Possibly, these proxy associations might vary by population, and hence inhibit generalizability of the model, hence calibration in other settings should be performed. By contrast, one cannot assume that fractures can be prevented by modifying such factors because they are not necessarily on the pathophysiological path. Furthermore, both diagnoses and medications were only included as dichotomous predictors. Hence, information, such as the timing of diagnoses or the dosing of medications, was not considered in the model. Therefore, medication in FREM_ver2_ should be regarded more as a proxy of diagnoses than as a measurement of pharmaceutical effect on the patient. Lastly, due to the relatively low number of HF events observed in the cohorts, the results for HF should be considered with caution, especially in the younger age group.

The study also has a number of strengths, which may add to the clinical utility of the new risk tool. First, the study design utilizing a large population-based registry cohort, which implies sufficient statistical power and the possibility to split into 3 cohorts to minimize risk of overfitting while identifying predictors of moderate strength. Additionally, this design indicates high generalizability and long availability of predictions data due to the unselected inclusion of all inhabitants in Denmark aged 45 or more. Second, the tool is optimized to identify high *imminent* fracture risk, especially for the older population (aged 65+), so that DXA resources can be appropriately targeted to those at the highest risk of fracture here and now. Moreover, the study utilizes a validated algorithm for identifying osteoporotic fractures from registry data, improving the validity of the outcome and hence of the prediction models.^18^ Importantly, the prediction models are structured to make them feasible for implementation in an automatic opportunistic screening program in general practice, utilizing predictors available in electronic health records, a possibility that is currently being evaluated in a pilot implementation study (CHOICE).

Furthermore, the applied LASSO logistic regression results in an explicit (log-linear) prediction model with odds ratios that can be directly clinically interpreted. This ensures both interpretability, as well as avoiding any concerns on separation between the (published) prediction model and the (highly confidential) training data, a concern that has been stated regarding more black-box machine learning models.^35^ Lastly, our supplementary analyses with 5-yr lookback, respectively, 2-yr imminent fracture risk (with both 5- and 15-yr lookback) predictions, show that FREM_ver2_ is robust toward different time frames for predictor identification as well as outcome.

## Conclusion

In conclusion, adding medication predictors marginally improved the predictive performance of the FREM_ver2_ model for MOF and did not enhance HF prediction. This indicated that medication as a dichotomous proxy for diagnoses only contributes with limited additional information in the Danish universal healthcare setting. However, stratifying the models and cut-offs by age groups substantially enhanced the balance between sensitivity and specificity, particularly in individuals aged 65+ yr, while not achieving a useful predictive power in individuals aged 45-64. This refinement may present FREM_ver2_ as a viable tool for opportunistic screening in older populations, despite limited improvements in overall prediction accuracy. However, currently FREM_ver2_ has only been validated in a Danish setting, and hence external validation in other locations and health care settings is necessary, before possible implementation for opportunistic screening outside of Denmark.

## Supplementary Material

FREM2_supplementary_final_adjustments_zjaf156

## Data Availability

The data underlying this article cannot be shared publicly due to the data-sharing regulations of the Danish Health Data Authority concerning individual data.^36^ Aggregated data (results) and do-files can be extracted and shared upon request by emailing the corresponding author.

## Notes

Ethics: Register-based studies do not require an ethics committee approval according to Danish law. The study was approved by the Danish Data Protection Agency (journal number: 2008-58-0035).
