# Supplementary material for: An enhanced Fracture Risk Evaluation Model (FREM) using national health data on morbidity and medications
Source: J Bone Miner Res. 2025 Oct 31;41(1):25–37. doi: 10.1093/jbmr/zjaf156 (PMC12765684; doi:10.1093/jbmr/zjaf156)

Supplemental Tables and Figures

Table S1: Exclusion criteria: Osteoporosis treatment and osteoporosis diagnosis

Name	ATC code (DNPR)	Procedures used in the Danish hospitals	ICD-10 code
**Osteoporosis treatment**			
Bisphosphonate	M05BA01, M05BA04, M05BA06, M05BA07, M05BA08, M05BB01, M05BB03,	BWHB40A, BWHB40B	
Strotium ranelate	M05BX03		
Denosumab	M05BX04	BWHB42	
Teriparatide	H05AA02		
Romosozumab	M05BX06		
Raloxifen	G03XC01		
**Osteoporosis diagnosis**			
			M80*, M81*

DNPR: Danish National Prescription Register, ICD: International Classification of Diseases
*including underlying categories of the codes

Table S2. Study population characteristics stratified by sex and cohorts

	Development cohort	Model validation cohort	Cutoff validation cohort	Development cohort	Model validation cohort	Cutoff validation cohort
		Females 45-64 years			Males 45-64 years	
		N= 731,707			*N= 759,329*	
	N= 439,024 (60.0%)	*N= 146,342* (20.0%)	*N= 146,341* (20.0%)	*N= 455,597* (60.0%)	*N=* 151,866 (20.0%)	*N=151,866 (20.0%)*
Age, years (median, (Q1; Q3))	55 (50;59)	55 (50;59)	55 (50;59)	55 (50;59)	55 (50;59)	55 (50;59)
Age categorized, N (%)						
45-49	114,986 (26.2%)	38,198 (26.1%)	38,392 (26.2%)	115,314 (25.3%)	38,587 (25.4%)	38,324 (25.2%)
50-54	113,530 (25.9%)	38,016 (26.0%)	37,555 (25.7%)	116,416 (25.5%)	38,675 (25.5%)	39,250 (25.8%)
55-59	115,788 (26.4%)	38,669 (26.6%)	38,692 (26.4%)	122,372 (26.9%)	40,433 (26.6%)	40,605 (26.7%)
60-64	94,720 (21.6%)	31,459 (21.5%)	31,702 (21.7%)	101,495 (22.3%)	34,171 (22.5%)	33,687 (22.2%)
Incident MOF in 2022, N (%)	2,751 (0.6%)	877 (0.6%)	983 (0.7%)	1,479 (0.3%)	474 (0.3%)	499 (0.3%)
45-49	254	71	80	294	79	82
50-54	543	175	189	331	107	119
55-59	936	319	348	438	149	143
60-64	1,018	312	366	416	139	155
Incident HF in 2022, N (%)	174 (0.0%)	66 (0.0%)	54 (0.0%)	221 (0.0%)	61 (0.0%)	66 (0.0%)
CCI grouped						
0	379,434 (86.4%)	126,511 (86.4%)	126,584 (86.5%)	401,536 (88.1%)	133,759 (88.1%)	134,012 (88.2%)
1-2	52,765 (12.0%)	17,563 (12.0%)	17,463 (11.9%)	46,394 (10.2%)	15,606 (10.3%)	15,417 (10.2%)
3+	6,825 (1.6%)	2,268 (1.5%)	2,294 (1.6%)	7,667 (1.7%)	2,501 (1.6%)	2,437 (1.6%)
Death 2022	1,247 (0.3%)	372 (0.3%)	391 (0.3%)	2,222 (0.5%)	723 (0.5%)	756 (0.5%)
Emigration 2022	784 (0.2%)	254 (0.2%)	242 (0.2%)	1,506 (0.3%)	496 (0.3%)	538 (0.4%)
Incomplete lookback	11,358 (2.6%)	3,683 (2.5%)	3,699 (2.5%)	16,384 (3.6%)	5,382 (3.5%)	5,250 (3.5%)
		Females 65+ years			Males 65+ years	
		N= 488,728			*N= 513,416*	
	N= 293,237 (60.0%)	*N= 97,745* (20.0%)	*N= 97,746* (20.0%)	*N= 308,050* (60.0%)	*N= 102,683* (20.0%)	*N= 102,683 (20.0%)*
Age, years (median, (Q1; Q3))	74 (69;80)	74 (69;80)	74 (69;80)	74 (69;79)	74 (69;79)	74 (69;79)
Age categorized, N (%)						
65-69	83,111 (28.3%)	27,733 (28.4%)	27,628 (28.3%)	90,805 (29.5%)	30,252 (29.5%)	30,081 (29.3%)
70-74	75,805 (25.9%)	25,231 (25.8%)	25,303 (25.9%)	83,107 (27.0%)	27,811 (27.1%)	27,439 (26.7%)
75-79	65,187 (22.2%)	21,452 (21.9%)	21,702 (22.2%)	71,206 (23.1%)	23,648 (23.0%)	24,015 (23.4%)
80.0+	69,134 (23.6%)	23,329 (23.9%)	23,113 (23.6%)	62,932 (20.4%)	20,972 (20.4%)	21,148 (20.6%)
Incident MOF in 2022, N (%)	6,462 (2.2%)	2,063 (2.1%)	2,048 (2.1%)	2,823 (0.9%)	942 (0.9%)	907 (0.9%)
65-69	1,128	377	374	482	155	151
70-74	1,336	404	441	548	218	156
75-79	1,401	464	413	666	219	215
80.0+	2,597	818	820	1,127	350	385

Incident HF in 2022, N (%)	1,966 (0.7%)	608 (0.6%)	625 (0.6%)	1,245 (0.4%)	406 (0.4%)	426 (0.4%)
CCI grouped						
0	212,770 (72.6%)	70,867 (72.5%)	71,193 (72.8%)	206,669 (67.1%)	68,643 (66.8%)	68,881 (67.1%)
1-2	64,606 (22.0%)	21,526 (22.0%)	21,357 (21.8%)	77,762 (25.2%)	26,087 (25.4%)	26,040 (25.4%)
3+	15,861 (5.4%)	5,352 (5.5%)	5,196 (5.3%)	23,619 (7.7%)	7,953 (7.7%)	7,762 (7.6%)
Death 2022	9,751 (3.3%)	3,190 (3.3%)	3,181 (3.3%)	12,776 (4.1%)	4,227 (4.1%)	4,201 (4.1%)
Emigration 2022	128 (0.0%)	58 (0.1%)	35 (0.0%)	186 (0.1%)	62 (0.1%)	54 (0.0%)
Incomplete lookback	2,054 (0.7%)	698 (0.7%)	727 (0.7%)	3,462 (1.1%)	1,173 (1.1%)	1,170 (1.1%)

MOF: Major Osteoporotic Fracture, HF: Hip Fracture

Table S3 Identified predictors for Major osteoporotic Fracture risk (A-D)

A: Females Aged 45-64 years

Risk factor	Name	Coefficient	Confidence interval		p-value	OR
ICD-10/ ATC code			Lower	Upper		
**Age**	45-49	Ref	-	-	-	1
	50-54	0,672	0,519	0,825	0,000	1,958
	55-59	1,097	0,951	1,244	0,000	2,995
	60-64	1,270	1,120	1,419	0,000	3,561
**Diagnosis**						
S42	Fracture of shoulder and upper arm	1,148	0,999	1,297	0,000	3,152
R45	Symptoms and signs involving emotional state	1,045	0,397	1,693	0,000	2,843
S52	Fracture of forearm	0,970	0,855	1,085	0,000	2,638
G25	Other extrapyramidal and movement disorders	0,852	0,353	1,351	0,000	2,344
S32	Fracture of lumbar spine and pelvis	0,806	0,501	1,111	0,000	2,239
S41	Open wound of shoulder and upper arm	0,804	0,189	1,419	0,010	2,234
H46	Optic neuritis	0,756	0,043	1,469	0,040	2,130
R43	Disturbances of smell and taste	0,750	-0,006	1,507	0,050	2,117
S22	Fracture of rib(s), sternum and thoracic spine	0,710	0,420	1,001	0,000	2,034
S76	Injury of muscle, fascia and tendon at hip and thigh level	0,701	0,084	1,317	0,030	2,016
H50	Other strabismus	0,688	0,295	1,081	0,000	1,990
Q82	Other congenital malformations of skin	0,621	0,106	1,137	0,020	1,861
D51	Vitamin B12 deficiency anaemia	0,589	-0,071	1,250	0,080	1,802
R25	Abnormal involuntary movements	0,528	0,209	0,847	0,000	1,696
N17	Acute renal failure	0,516	0,012	1,021	0,040	1,675
D33	Benign neoplasm of meninges	0,515	-0,091	1,121	0,100	1,674
S72	Fracture of femur	0,485	0,081	0,888	0,020	1,624
E68	Sequelae of hyperalimentation	0,481	0,164	0,798	0,000	1,618
F60	Specific personality disorders	0,476	-0,146	1,099	0,130	1,610
A04	Other bacterial intestinal infections	0,404	-0,085	0,892	0,110	1,498
S92	Fracture of foot and toe, except ankle	0,401	0,255	0,548	0,000	1,493
S62	Fracture at wrist and hand level	0,396	0,251	0,541	0,000	1,486
K05	Gingivitis and periodontal diseases	0,388	-0,074	0,849	0,100	1,474
R59	Enlarged lymph nodes	0,381	-0,036	0,797	0,070	1,464
I67	Other cerebrovascular diseases	0,379	-0,080	0,837	0,110	1,461

K12	Stomatitis and related lesions	0,359	-0,188	0,906	0,200	1,432
G63	Polyneuropathy in diseases classified elsewhere	0,353	-0,316	1,022	0,300	1,423
I26	Pulmonary embolism	0,348	-0,032	0,729	0,070	1,416
R18	Ascites	0,347	-0,295	0,989	0,290	1,415
M13	Other arthritis	0,339	0,043	0,635	0,020	1,404
K76	Other diseases of liver	0,322	0,007	0,637	0,050	1,380
J96	Respiratory failure, not elsewhere classified	0,316	-0,016	0,648	0,060	1,372
C34	Malignant neoplasm of bronchus and lung	0,307	-0,209	0,822	0,240	1,359
S66	Injury of muscle, fascia and tendon at wrist and hand level	0,300	-0,023	0,623	0,070	1,350
E83	Disorders of mineral metabolism	0,291	-0,194	0,775	0,240	1,338
R74	Abnormal serum enzyme levels	0,270	-0,150	0,691	0,210	1,310
H33	Retinal detachments and breaks	0,255	-0,116	0,626	0,180	1,290
D27	Benign neoplasm of ovary	0,250	-0,021	0,522	0,070	1,284
I35	Nonrheumatic aortic valve disorders	0,243	-0,161	0,646	0,240	1,275
R60	Edema, not elsewhere classified	0,241	-0,148	0,631	0,220	1,273
C50	Malignant neoplasm of breast	0,233	0,003	0,463	0,050	1,262
M18	Osteoarthritis of first carpometacarpal joint	0,226	-0,049	0,501	0,110	1,254
K56	Paralytic ileus and intestinal obstruction without hernia	0,215	-0,143	0,572	0,240	1,240
S63	Dislocation and sprain of joints and ligaments at wrist and hand level	0,213	0,057	0,369	0,010	1,237
M85	Other disorders of bone density and structure	0,212	0,012	0,411	0,040	1,236
R11	Nausea and vomiting	0,211	-0,075	0,496	0,150	1,235
S82	Fracture of lower leg, including ankle	0,210	0,047	0,374	0,010	1,234
G40	Epilepsy	0,205	-0,097	0,507	0,180	1,228
S01	Open wound of head	0,199	0,037	0,361	0,020	1,220
G45	Transient cerebral ischemic attacks and related syndromes	0,195	-0,106	0,497	0,200	1,215
E10	Type 1 diabetes mellitus	0,193	-0,131	0,516	0,240	1,213
M67	Other disorders of synovium and tendon	0,192	0,006	0,378	0,040	1,212
K70	Alcoholic liver disease	0,184	-0,315	0,682	0,470	1,202
H53	Visual disturbances	0,177	-0,145	0,500	0,280	1,194
F10	Mental and behavioural disorders due to use of alcohol	0,175	-0,069	0,420	0,160	1,191
J15	Bacterial pneumonia, not elsewhere classified	0,155	-0,167	0,476	0,350	1,168

S30	Superficial injury of abdomen, lower back, pelvis and external genitals	0,146	-0,105	0,398	0,250	1,157
E87	Other disorders of fluid, electrolyte and acid-base balance	0,133	-0,153	0,420	0,360	1,142
S80	Superficial injury of knee and lower leg	0,132	-0,030	0,295	0,110	1,141
I63	Cerebral infarction	0,121	-0,159	0,400	0,400	1,129
H25	Age-related cataract	0,111	-0,126	0,347	0,360	1,117
D64	Other anaemias	0,104	-0,166	0,373	0,450	1,110
S00	Superficial injury of head	0,091	-0,100	0,282	0,350	1,095
N92	Excessive, frequent and irregular menstruation	-0,170	-0,295	-0,045	0,010	0,844
M51	Thoracic, thoracolumbar, and lumbosacral intervertebral disc disorders	-0,187	-0,354	-0,020	0,030	0,829
M50	Cervical disc disorders	-0,261	-0,514	-0,008	0,040	0,770
O72	Postpartum hemorrhage	-0,346	-0,680	-0,013	0,040	0,708
T07	Unspecified multiple injuries	-0,612	-1,170	-0,055	0,030	0,542
M84	Disorder of continuity of bone	-0,615	-1,374	0,143	0,110	0,541
F41	Other anxiety disorders	-0,660	-1,105	-0,214	0,000	0,517
K50	Crohn's disease	-0,686	-1,318	-0,054	0,030	0,504
L73	Other follicular disorders	-0,760	-1,511	-0,008	0,050	0,468
S27	Injury of other and unspecified intrathoracic organs	-0,806	-1,665	0,053	0,070	0,447
G50	Disorders of trigeminal nerve	-0,897	-1,707	-0,087	0,030	0,408
J20	Acute bronchitis	-0,947	-1,841	-0,052	0,040	0,388
E27	Other disorders of adrenal gland	-0,969	-1,965	0,026	0,060	0,379
T40	Poisoning by, adverse effect of and underdosing of narcotics and psychodysleptics	-1,140	-1,974	-0,306	0,010	0,320
F31	Bipolar disorder	-1,303	-2,326	-0,279	0,010	0,272
J22	Unspecified acute lower respiratory infection	-1,395	-2,825	0,034	0,060	0,248
T13	Other injuries of lower limb, level unspecified	-2,024	-3,991	-0,058	0,040	0,132
**Medicine**						
A09	Digestives, incl. enzymes	0,540	0,002	1,079	0,050	1,716
H04	Pancreatic hormones	0,433	-0,120	0,985	0,130	1,542
N07	Other nervous system drugs	0,265	0,148	0,381	0,000	1,303
A06	Drugs for constipation	0,140	0,025	0,255	0,020	1,150
N03	Antiepileptics	0,097	-0,068	0,261	0,250	1,102
A07	Antidiarrheals, intenstinal antiinflammatory/antiinfective agents	0,090	-0,035	0,216	0,160	1,094

N02	Analgetics	0,080	-0,014	0,174	0,100	1,083
N06	Psychoanaleptics	0,075	-0,012	0,163	0,090	1,078
B03	Antianemic preparation	0,037	-0,076	0,150	0,520	1,038
A12	Mineral supplements	0,010	-0,118	0,138	0,880	1,010
G02	Other gynelogicals	-0,127	-0,234	-0,020	0,020	0,881
C05	Vasoprotectives	-0,137	-0,224	-0,050	0,000	0,872
	Baseline risk (constant term)	-6,285	-6,435	-6,135	0,000	

B: Males Aged 45-64 years

Risk factor	Name	Coefficient	Confidence interval		p-value	OR
ICD-10/ ATC code			Lower	Upper		
**Age**	45-49	Ref	-	-	-	1
	50-54	0,061	-0,098	0,219	0,450	1,063
	55-59	0,229	0,079	0,380	0,000	1,257
	60-64	0,292	0,136	0,448	0,000	1,339
**Diagnosis**						
B02	Zoster	1,246	0,658	1,834	0,000	3,476
S12	Fracture of cervical vertebra and other parts of neck	0,987	0,547	1,426	0,000	2,683
S42	Fracture of shoulder and upper arm	0,835	0,649	1,022	0,000	2,305
C20	Malignant neoplasm of rectum	0,796	0,086	1,507	0,030	2,217
D09	Carcinoma in situ of other and unspecified sites	0,782	-0,033	1,598	0,060	2,186
G35	Multiple sclerosis	0,721	0,144	1,298	0,010	2,056
S72	Fracture of femur	0,704	0,360	1,047	0,000	2,022
S52	Fracture of forearm	0,701	0,509	0,894	0,000	2,016
B23	Human immunodeficiency virus [HIV] disease resulting in other conditions	0,660	0,045	1,275	0,040	1,935
I67	Other cerebrovascular diseases	0,645	0,031	1,259	0,040	1,906
C61	Malignant neoplasm of prostate	0,605	0,144	1,065	0,010	1,831
H47	Other disorders of optic [2nd] nerve and visual pathways	0,595	-0,203	1,393	0,140	1,813
E27	Other disorders of adrenal gland	0,572	-0,196	1,340	0,140	1,772
F10	Mental and behavioural disorders due to use of alcohol	0,556	0,342	0,771	0,000	1,744
G25	Other extrapyramidal and movement disorders	0,547	-0,151	1,245	0,120	1,728
S32	Fracture of lumbar spine and pelvis	0,547	0,213	0,882	0,000	1,728
K52	Other noninfective gastroenteritis and colitis	0,541	0,199	0,884	0,000	1,718
S82	Fracture of lower leg, including ankle	0,530	0,323	0,738	0,000	1,699
N17	Acute renal failure	0,518	-0,009	1,044	0,050	1,679
B18	Chronic viral hepatitis	0,515	0,098	0,932	0,020	1,674
S22	Fracture of rib(s), sternum and thoracic spine	0,499	0,244	0,754	0,000	1,647
K65	Peritonitis	0,476	-0,209	1,160	0,170	1,610
D69	Purpura and other haemorrhagic conditions	0,472	-0,206	1,150	0,170	1,603
R41	Other symptoms and signs involving cognitive functions and awareness	0,459	0,012	0,907	0,040	1,582

H36	Retinal disorders in diseases classified elsewhere	0,458	0,025	0,891	0,040	1,581
T79	Certain early complications of trauma, not elsewhere classified	0,436	-0,028	0,900	0,070	1,547
H25	Age-related cataract	0,427	0,133	0,721	0,000	1,533
E16	Other disorders of pancreatic internal secretion	0,386	-0,185	0,956	0,180	1,471
E83	Disorders of mineral metabolism	0,383	-0,223	0,988	0,220	1,467
S62	Fracture at wrist and hand level	0,365	0,199	0,532	0,000	1,441
F33	Recurrent depressive disorder	0,359	-0,089	0,808	0,120	1,432
K22	Other diseases of oesophagus	0,354	-0,056	0,764	0,090	1,425
I95	Hypotension	0,346	-0,236	0,928	0,240	1,413
M85	Other disorders of bone density and structure	0,344	-0,147	0,835	0,170	1,411
K26	Duodenal ulcer	0,332	-0,271	0,935	0,280	1,394
G40	Epilepsy	0,307	-0,048	0,662	0,090	1,359
S02	Fracture of skull and facial bones	0,301	0,016	0,587	0,040	1,351
R25	Abnormal involuntary movements	0,268	-0,088	0,625	0,140	1,307
M72	Fibroblastic disorders	0,254	-0,062	0,570	0,120	1,289
T14	Injury of unspecified body region	0,250	0,034	0,467	0,020	1,284
I73	Other peripheral vascular diseases	0,225	-0,171	0,621	0,260	1,252
S93	Dislocation and sprain of joints and ligaments at ankle, foot and toe level	0,225	0,051	0,400	0,010	1,252
R55	Syncope and collapse	0,215	-0,021	0,452	0,070	1,240
S20	Superficial injury of thorax	0,213	0,000	0,426	0,050	1,237
E10	Type 1 diabetes mellitus	0,177	-0,222	0,576	0,390	1,194
F17	Mental and behavioural disorders due to use of tobacco	0,166	-0,126	0,458	0,260	1,181
S00	Superficial injury of head	0,147	-0,063	0,357	0,170	1,158
I85	Esophageal varices	0,142	-0,543	0,827	0,680	1,153
I63	Cerebral infarction	0,128	-0,168	0,424	0,400	1,137
S92	Fracture of foot and toe, except ankle	0,121	-0,104	0,346	0,290	1,129
T93	Sequelae of injuries of lower limb	0,120	-0,132	0,372	0,350	1,127
R56	Convulsions, not elsewhere classified	0,119	-0,289	0,527	0,570	1,126
S80	Superficial injury of knee and lower leg	0,117	-0,100	0,333	0,290	1,124
T84	Complications of internal orthopedic prosthetic devices, implants and grafts	0,106	-0,227	0,439	0,530	1,112
J44	Other chronic obstructive pulmonary disease	0,100	-0,209	0,409	0,530	1,105
R29	Other symptoms and signs involving the nervous and musculoskeletal systems	0,089	-0,148	0,326	0,460	1,093

R53	Malaise and fatigue	0,087	-0,203	0,377	0,560	1,091
S01	Open wound of head	0,074	-0,089	0,237	0,370	1,077
K92	Other diseases of digestive system	0,062	-0,330	0,454	0,760	1,064
T42	Poisoning by, adverse effect of and underdosing of antiepileptic, sedative- hypnotic and antiparkinsonism drugs	-0,499	-1,300	0,301	0,220	0,607
F12	Cannabis related disorders	-0,525	-1,477	0,426	0,280	0,592
T50	Poisoning by, adverse effect of and underdosing of diuretics and other and unspecified drugs, medicaments and biological substances	-0,607	-1,310	0,096	0,090	0,545
S27	Injury of other and unspecified intrathoracic organs	-0,619	-1,198	-0,040	0,040	0,538
S36	Injury of intra-abdominal organs	-0,728	-1,761	0,305	0,170	0,483
F11	Opioid related disorders	-0,848	-1,755	0,060	0,070	0,428
T65	Toxic effect of other and unspecified substances	-0,870	-1,795	0,056	0,070	0,419
E13	Other specified diabetes mellitus	-1,256	-2,306	-0,206	0,020	0,285
K50	Crohn's disease	-1,480	-2,873	-0,087	0,040	0,228
S21	Open wound of thorax	-1,521	-3,509	0,467	0,130	0,218
I71	Aortic aneurysm and dissection	-1,788	-3,187	-0,388	0,010	0,167
**Medicine**						
H04	Pancreatic hormones	0,359	-0,256	0,974	0,250	1,432
A06	Drugs for constipation	0,215	0,059	0,370	0,010	1,240
N03	Antiepileptics	0,201	-0,022	0,425	0,080	1,223
B03	Antianemic preparations	0,165	-0,013	0,343	0,070	1,179
J01	Antibacterials for systemic use	0,154	-0,019	0,328	0,080	1,166
N02	Analgetics	0,138	0,015	0,261	0,030	1,148
N07	Other nervous system drugs	0,125	-0,029	0,280	0,110	1,133
N05	Psycholeptics	0,048	-0,087	0,182	0,490	1,049
N06	Psychoanaleptics	0,030	-0,105	0,166	0,660	1,030
A12	Mineral supplements	0,011	-0,186	0,208	0,920	1,011
	Baseline risk (constant term)	-6,580	-6,775	-6,385	0,000	

C: Females Aged 65+ years

Risk factor	Name	Coefficient	Confidence interval		p-value	OR
ICD-10/ ATC code			Lower	Upper		
**Age**	65-69	Ref	-	-	-	1
	70-74	0,249	0,169	0,330	0,000	1,283
	75-79	0,413	0,332	0,495	0,000	1,511
	80+	0,860	0,781	0,939	0,000	2,363
**Diagnosis**						
S42	Fracture of shoulder and upper arm	0,738	0,657	0,818	0,000	2,092
H54	Blindness and low vision	0,570	0,269	0,870	0,000	1,768
G63	Polyneuropathy in diseases classified elsewhere	0,559	0,201	0,916	0,000	1,749
C79	Secondary malignant neoplasm of other and unspecified sites	0,541	0,075	1,006	0,020	1,718
S32	Fracture of lumbar spine and pelvis	0,531	0,388	0,675	0,000	1,701
R18	Ascites	0,525	0,098	0,952	0,020	1,690
T11	Other injuries of upper limb, level unspecified	0,511	0,072	0,951	0,020	1,667
J10	Influenza due to other identified influenza virus	0,503	0,020	0,987	0,040	1,654
S52	Fracture of forearm	0,473	0,406	0,541	0,000	1,605
S33	Dislocation and sprain of joints and ligaments of lumbar spine and pelvis	0,458	0,079	0,838	0,020	1,581
K41	Femoral hernia	0,427	0,014	0,840	0,040	1,533
S22	Fracture of rib(s), sternum and thoracic spine	0,427	0,228	0,626	0,000	1,533
T82	Complications of cardiac and vascular prosthetic devices, implants and grafts	0,418	-0,111	0,946	0,120	1,519
G20	Parkinson disease	0,415	0,161	0,668	0,000	1,514
G31	Other degenerative diseases of nervous system, not elsewhere classified	0,379	0,027	0,731	0,030	1,461
T50	Poisoning by, adverse effect of and underdosing of diuretics and other and unspecified drugs, medicaments and biological substances	0,368	-0,040	0,777	0,080	1,445
F00	Dementia in Alzheimer disease	0,364	0,189	0,540	0,000	1,439
T51	Toxic effect of alcohol	0,355	-0,166	0,876	0,180	1,426
R17	Hyperbilirubinæmi med eller uden gulsot IKA	0,352	-0,144	0,849	0,160	1,422

T79	Certain early complications of trauma, not elsewhere classified	0,333	0,098	0,567	0,010	1,395
R58	Hemorrhage, not elsewhere classified	0,331	-0,110	0,772	0,140	1,392
L50	Urticaria	0,327	0,069	0,584	0,010	1,387
T18	Foreign body in alimentary tract	0,323	-0,005	0,652	0,050	1,381
J43	Emphysema	0,318	-0,012	0,649	0,060	1,374
J13	Pneumonia due to Streptococcus pneumoniae	0,300	-0,172	0,773	0,210	1,350
L03	Cellulitis and acute lymphangitis	0,282	-0,034	0,597	0,080	1,326
G30	Alzheimer disease	0,277	0,099	0,455	0,000	1,319
H16	Keratitis	0,276	-0,044	0,597	0,090	1,318
S62	Fracture at wrist and hand level	0,259	0,157	0,361	0,000	1,296
H34	Retinal vascular occlusions	0,255	0,046	0,464	0,020	1,290
S30	Superficial injury of abdomen, lower back, pelvis and external genitals	0,254	0,082	0,425	0,000	1,289
D51	Vitamin B12 deficiency anaemia	0,250	-0,210	0,711	0,290	1,284
G50	Disorders of trigeminal nerve	0,248	-0,089	0,584	0,150	1,281
G25	Other extrapyramidal and movement disorders	0,233	-0,063	0,529	0,120	1,262
C20	Malignant neoplasm of rectum	0,223	-0,051	0,498	0,110	1,250
K86	Other diseases of pancreas	0,219	-0,095	0,534	0,170	1,245
K55	Vascular disorders of intestine	0,213	-0,135	0,561	0,230	1,237
G91	Hydrocephalus	0,205	-0,165	0,575	0,280	1,228
K22	Other diseases of oesophagus	0,199	-0,025	0,424	0,080	1,220
S82	Fracture of lower leg, including ankle	0,194	0,092	0,296	0,000	1,214
R15	Fecal incontinence	0,193	-0,018	0,404	0,070	1,213
F10	Mental and behavioural disorders due to use of alcohol	0,189	0,006	0,372	0,040	1,208
I51	Complications and ill-defined descriptions of heart disease	0,188	-0,077	0,453	0,160	1,207
S20	Superficial injury of thorax	0,186	0,029	0,342	0,020	1,204
S92	Fracture of foot and toe, except ankle	0,181	0,059	0,302	0,000	1,198
M06	Other rheumatoid arthritis	0,175	-0,044	0,395	0,120	1,191
R04	Hemorrhage from respiratory passages	0,174	0,026	0,322	0,020	1,190
R63	Symptoms and signs concerning food and fluid intake	0,154	-0,045	0,354	0,130	1,166
E10	Type 1 diabetes mellitus	0,153	-0,028	0,334	0,100	1,165
S90	Superficial injury of ankle, foot and toes	0,150	0,004	0,295	0,040	1,162
T93	Sequelae of injuries of lower limb	0,149	-0,008	0,305	0,060	1,161

J90	Pleural effusion, not elsewhere classified	0,146	-0,135	0,426	0,310	1,157
A49	Bacterial infection of unspecified site	0,139	-0,015	0,294	0,080	1,149
S01	Open wound of head	0,137	0,043	0,230	0,000	1,147
S63	Dislocation and sprain of joints and ligaments at wrist and hand level	0,130	0,000	0,261	0,050	1,139
G40	Epilepsy	0,128	-0,093	0,349	0,260	1,137
S93	Dislocation and sprain of joints and ligaments at ankle, foot and toe level	0,117	0,006	0,228	0,040	1,124
D50	Iron deficiency anaemia	0,116	-0,060	0,292	0,200	1,123
F32	Depressive episode	0,115	-0,083	0,314	0,250	1,122
R53	Malaise and fatigue	0,114	-0,008	0,236	0,070	1,121
R91	Abnormal findings on diagnostic imaging of lung	0,102	-0,025	0,230	0,110	1,107
S40	Superficial injury of shoulder and upper arm	0,101	-0,041	0,244	0,160	1,106
R25	Abnormal involuntary movements	0,095	-0,161	0,351	0,470	1,100
H91	Other and unspecified hearing loss	0,094	0,016	0,171	0,020	1,099
S80	Superficial injury of knee and lower leg	0,084	-0,035	0,203	0,170	1,088
T88	Other complications of surgical and medical care, not elsewhere classified	0,081	-0,068	0,230	0,290	1,084
R41	Other symptoms and signs involving cognitive functions and awareness	0,077	-0,072	0,227	0,310	1,080
S60	Superficial injury of wrist, hand and fingers	0,074	-0,036	0,184	0,190	1,077
E87	Other disorders of fluid, electrolyte and acid-base balance	0,071	-0,049	0,191	0,250	1,074
I25	Chronic ischemic heart disease	0,070	-0,039	0,179	0,210	1,073
H26	Other cataract	0,068	-0,027	0,162	0,160	1,070
R55	Syncope and collapse	0,067	-0,032	0,165	0,180	1,069
S72	Fracture of femur	0,067	-0,063	0,197	0,310	1,069
H35	Other retinal disorders	0,066	-0,027	0,159	0,160	1,068
J44	Other chronic obstructive pulmonary disease	0,050	-0,061	0,162	0,380	1,051
S70	Superficial injury of hip and thigh	0,042	-0,094	0,178	0,550	1,043
D64	Other anaemias	0,041	-0,093	0,175	0,550	1,042
M75	Shoulder lesions	-0,085	-0,182	0,013	0,090	0,919
G56	Mononeuropathies of upper limb	-0,094	-0,211	0,024	0,120	0,910
M17	Osteoarthritis of knee	-0,125	-0,202	-0,049	0,000	0,882
E66	Obesity	-0,139	-0,252	-0,027	0,010	0,870
I80	Phlebitis and thrombophlebitis	-0,174	-0,339	-0,010	0,040	0,840

H93	Other disorders of ear, not elsewhere classified	-0,219	-0,389	-0,050	0,010	0,803
N64	Other disorders of breast	-0,228	-0,501	0,045	0,100	0,796
M21	Other acquired deformities of limbs	-0,229	-0,461	0,004	0,050	0,795
H53	Visual disturbances	-0,240	-0,475	-0,005	0,050	0,787
E21	Hyperparathyroidism and other disorders of parathyroid gland	-0,255	-0,556	0,046	0,100	0,775
N84	Polyp of female genital tract	-0,259	-0,427	-0,091	0,000	0,772
K81	Cholecystitis	-0,268	-0,539	0,003	0,050	0,765
J96	Respiratory failure, not elsewhere classified	-0,280	-0,492	-0,069	0,010	0,756
M16	Coxarthrosis [arthrosis of hip]	-0,294	-0,389	-0,200	0,000	0,745
R33	Retention of urine	-0,299	-0,536	-0,062	0,010	0,742
M24	Other specific joint derangements	-0,302	-0,681	0,077	0,120	0,739
G44	Other headache syndromes	-0,322	-0,633	-0,011	0,040	0,725
H52	Disorders of refraction and accommodation	-0,337	-0,641	-0,032	0,030	0,714
G57	Mononeuropathies of lower limb	-0,339	-0,703	0,026	0,070	0,712
K58	Irritable bowel syndrome	-0,342	-0,595	-0,089	0,010	0,710
T07	Unspecified multiple injuries	-0,343	-0,812	0,126	0,150	0,710
S02	Fracture of skull and facial bones	-0,352	-0,601	-0,103	0,010	0,703
E83	Disorders of mineral metabolism	-0,374	-0,714	-0,034	0,030	0,688
F43	Reaction to severe stress, and adjustment disorders	-0,396	-0,777	-0,016	0,040	0,673
M66	Spontaneous rupture of synovium and tendon	-0,420	-0,837	-0,004	0,050	0,657
D62	Acute posthemorrhagic anemia	-0,426	-0,804	-0,049	0,030	0,653
J34	Other and unspecified disorders of nose and nasal sinuses	-0,428	-0,942	0,085	0,100	0,652
E89	Postprocedural endocrine and metabolic complications and disorders, not elsewhere classified	-0,436	-0,937	0,065	0,090	0,647
K08	Other disorders of teeth and supporting structures	-0,448	-0,865	-0,030	0,040	0,639
L97	Ulcer of lower limb, not elsewhere classified	-0,493	-0,839	-0,146	0,010	0,611
I24	Other acute ischemic heart diseases	-0,508	-1,141	0,125	0,120	0,602
S27	Injury of other and unspecified intrathoracic organs	-0,639	-1,293	0,014	0,060	0,528
G90	Disorders of autonomic nervous system	-0,701	-1,455	0,052	0,070	0,496
D86	Sarcoidosis	-0,723	-1,425	-0,021	0,040	0,485
K46	Unspecified abdominal hernia	-0,745	-1,633	0,143	0,100	0,475
T43	Poisoning by psychotropic drugs, not elsewhere classified	-0,909	-1,737	-0,082	0,030	0,403
H27	Other disorders of lens	-1,094	-2,237	0,049	0,060	0,335

T00	Superficial injuries involving multiple body regions	-1,454	-2,849	-0,058	0,040	0,234
**Medicine**						
A05	Bile and liver therapy	0,593	0,030	1,157	0,040	1,809
H04	Pancreatic hormones	0,405	0,023	0,788	0,040	1,499
A09	Digestives, incl. enzymes	0,281	-0,106	0,667	0,150	1,324
A04	Antiemetics and antinauseants	0,124	-0,006	0,254	0,060	1,132
N02	Analgetics	0,124	0,052	0,196	0,000	1,132
N06	Psychoanaleptics	0,121	0,063	0,180	0,000	1,129
N07	Other nervous system drugs	0,110	0,017	0,204	0,020	1,116
A06	Drugs for constipation	0,088	0,024	0,153	0,010	1,092
N03	Antiepileptics	0,078	-0,050	0,206	0,230	1,081
B03	Antianemic preparations	0,068	0,000	0,137	0,050	1,070
B01	Antithrombotic agents	0,045	-0,012	0,103	0,120	1,046
A12	Mineral supplements	0,028	-0,033	0,090	0,370	1,028
J05	Antivirals for systemic use	-0,071	-0,140	-0,001	0,050	0,931
D01	Antifungals for dermatological use	-0,079	-0,132	-0,027	0,000	0,924
C09	Agents acting on the renin-angiotensin system	-0,094	-0,148	-0,040	0,000	0,910
P03	Ectoparasiticides, incl. Scabicides, insecticides and repellents	-0,488	-0,873	-0,103	0,010	0,614
	Baseline risk (constant term)	-4,547	-4,629	-4,465	0,000	

D: Males Aged 65+ years

Risk factor	Name	Coefficient	Confidence interval		p-value	OR
**ICD-10/ ATC code**			**Lower**	**Upper**		
**Age**	65-69	Ref	-	-	-	1
	70-74	0,194	0,070	0,318	0,000	1,214
	75-79	0,488	0,365	0,610	0,000	1,629
	80+	1,008	0,889	1,128	0,000	2,740
**Diagnosis**						
B23	Human immunodeficiency virus [HIV] disease resulting in other conditions	1,134	0,460	1,808	0,000	3,108
C90	Multiple myeloma and malignant plasma cell neoplasms	0,999	0,446	1,552	0,000	2,716
T30	Burn and corrosion, body region unspecified	0,972	0,258	1,685	0,010	2,643
S12	Fracture of cervical vertebra and other parts of neck	0,877	0,488	1,266	0,000	2,404
S42	Fracture of shoulder and upper arm	0,877	0,732	1,023	0,000	2,404
L29	Pruritus	0,805	0,323	1,287	0,000	2,237
D46	Myelodysplastic syndromes	0,786	0,173	1,398	0,010	2,195
G60	Hereditary and idiopathic neuropathy	0,705	0,028	1,383	0,040	2,024
T59	Toxic effect of other gases, fumes and vapors	0,685	0,001	1,369	0,050	1,984
L73	Other follicular disorders	0,681	0,003	1,359	0,050	1,976
J14	Pneumonia due to Haemophilus influenzae	0,624	0,006	1,241	0,050	1,866
C88	Malignant immunoproliferative diseases	0,600	0,018	1,182	0,040	1,822
D32	Benign neoplasm of meninges	0,575	0,063	1,087	0,030	1,777
S72	Fracture of femur	0,567	0,383	0,751	0,000	1,763
S52	Fracture of forearm	0,564	0,402	0,726	0,000	1,758
R63	Symptoms and signs concerning food and fluid intake	0,534	0,303	0,766	0,000	1,706
S32	Fracture of lumbar spine and pelvis	0,492	0,262	0,723	0,000	1,636
I61	Intracerebral haemorrhage	0,464	0,189	0,739	0,000	1,590
D45	Polycythaemia vera	0,457	-0,182	1,097	0,160	1,579
M21	Other acquired deformities of limbs	0,457	0,147	0,766	0,000	1,579
G31	Other degenerative diseases of nervous system, not elsewhere classified	0,444	0,092	0,796	0,010	1,559
T79	Certain early complications of trauma, not elsewhere classified	0,432	0,162	0,702	0,000	1,540
C91	Lymphoid leukemia	0,429	0,028	0,831	0,040	1,536
C34	Malignant neoplasm of bronchus and lung	0,426	0,142	0,710	0,000	1,531

J86	Pyothorax	0,408	-0,092	0,908	0,110	1,504
N99	Postprocedural disorders of genitourinary system, not elsewhere classified	0,404	-0,086	0,894	0,110	1,498
A08	Viral and other specified intestinal infections	0,403	-0,186	0,992	0,180	1,496
F00	Dementia in Alzheimer disease	0,400	0,124	0,676	0,000	1,492
J91	Pleural effusion in conditions classified elsewhere	0,379	-0,173	0,931	0,180	1,461
E16	Other disorders of pancreatic internal secretion	0,376	0,011	0,741	0,040	1,456
K27	Peptic ulcer, site unspecified	0,376	-0,096	0,847	0,120	1,456
I87	Other disorders of veins	0,371	-0,040	0,781	0,080	1,449
F10	Mental and behavioural disorders due to use of alcohol	0,348	0,177	0,519	0,000	1,416
M06	Other rheumatoid arthritis	0,320	-0,062	0,702	0,100	1,377
I15	Secondary hypertension	0,319	-0,029	0,666	0,070	1,376
S82	Fracture of lower leg, including ankle	0,312	0,123	0,500	0,000	1,366
K76	Other diseases of liver	0,310	-0,033	0,654	0,080	1,363
B02	Zoster	0,302	-0,139	0,743	0,180	1,353
G91	Hydrocephalus	0,300	-0,090	0,690	0,130	1,350
M05	Seropositive rheumatoid arthritis	0,299	-0,119	0,717	0,160	1,349
G20	Parkinson disease	0,294	-0,004	0,593	0,050	1,342
F17	Mental and behavioural disorders due to use of tobacco	0,290	0,051	0,530	0,020	1,336
D23	Other benign neoplasms of skin	0,279	0,028	0,530	0,030	1,322
K85	Acute pancreatitis	0,276	-0,032	0,584	0,080	1,318
D63	Acute posthemorrhagic anemia	0,271	-0,281	0,823	0,340	1,311
K02	Dental caries	0,271	-0,167	0,709	0,230	1,311
K74	Fibrosis and cirrhosis of liver	0,259	-0,239	0,757	0,310	1,296
N21	Calculus of lower urinary tract	0,247	-0,074	0,568	0,130	1,280
N18	Chronic kidney disease	0,234	0,061	0,407	0,010	1,264
R53	Malaise and fatigue	0,227	0,068	0,386	0,010	1,255
S62	Fracture at wrist and hand level	0,227	0,062	0,392	0,010	1,255
D50	Iron deficiency anaemia	0,220	-0,019	0,458	0,070	1,246
I63	Cerebral infarction	0,217	0,090	0,344	0,000	1,242
S63	Dislocation and sprain of joints and ligaments at wrist and hand level	0,216	0,004	0,428	0,050	1,241
K62	Other diseases of anus and rectum	0,213	0,039	0,387	0,020	1,237
G30	Alzheimer disease	0,208	-0,079	0,495	0,160	1,231
S93	Dislocation and sprain of joints and ligaments at ankle, foot and toe level	0,202	-0,013	0,416	0,070	1,224

S90	Superficial injury of ankle, foot and toes	0,194	-0,047	0,435	0,110	1,214
K04	Diseases of pulp and periapical tissues	0,192	-0,129	0,514	0,240	1,212
S60	Superficial injury of wrist, hand and fingers	0,190	0,014	0,366	0,030	1,209
H36	Retinal disorders in diseases classified elsewhere	0,189	-0,080	0,459	0,170	1,208
J44	Other chronic obstructive pulmonary disease	0,188	0,048	0,328	0,010	1,207
D47	Other neoplasms of uncertain behavior of lymphoid, hematopoietic and related tissue	0,181	-0,142	0,505	0,270	1,198
K22	Other diseases of oesophagus	0,179	-0,066	0,424	0,150	1,196
J90	Pleural effusion, not elsewhere classified	0,167	-0,118	0,453	0,250	1,182
S22	Fracture of rib(s), sternum and thoracic spine	0,155	-0,056	0,366	0,150	1,168
A46	Erysipelas	0,154	-0,026	0,334	0,090	1,166
L02	Cutaneous abscess, furuncle and carbuncle	0,154	-0,065	0,373	0,170	1,166
R41	Other symptoms and signs involving cognitive functions and awareness	0,146	-0,051	0,343	0,150	1,157
K05	Gingivitis and periodontal diseases	0,144	-0,195	0,483	0,410	1,155
S30	Superficial injury of abdomen, lower back, pelvis and external genitals	0,144	-0,128	0,417	0,300	1,155
R04	Hemorrhage from respiratory passages	0,137	-0,038	0,313	0,130	1,147
S01	Open wound of head	0,135	0,013	0,257	0,030	1,145
C61	Malignant neoplasm of prostate	0,134	-0,007	0,275	0,060	1,143
K59	Other functional intestinal disorders	0,119	-0,028	0,267	0,110	1,126
D64	Other anaemias	0,118	-0,045	0,281	0,150	1,125
I95	Hypotension	0,118	-0,121	0,356	0,330	1,125
E14	Unspecified diabetes mellitus	0,102	-0,132	0,336	0,390	1,107
G40	Epilepsy	0,101	-0,133	0,334	0,400	1,106
S00	Superficial injury of head	0,101	-0,076	0,278	0,260	1,106
K70	Alcoholic liver disease	0,089	-0,289	0,467	0,640	1,093
S20	Superficial injury of thorax	0,084	-0,116	0,284	0,410	1,088
S06	Intracranial injury	0,065	-0,110	0,240	0,460	1,067
E87	Other disorders of fluid, electrolyte and acid-base balance	0,058	-0,120	0,236	0,520	1,060
K92	Other diseases of digestive system	0,055	-0,171	0,280	0,630	1,057
R29	Other symptoms and signs involving the nervous and musculoskeletal systems	0,053	-0,079	0,184	0,430	1,054
H25	Age-related cataract	0,040	-0,053	0,133	0,400	1,041
J18	Pneumonia, organism unspecified	0,015	-0,105	0,136	0,810	1,015

M75	Shoulder lesions	-0,070	-0,210	0,070	0,330	0,932
D12	Benign neoplasm of colon, rectum, anus and anal canal	-0,139	-0,254	-0,024	0,020	0,870
R10	Abdominal and pelvic pain	-0,152	-0,277	-0,026	0,020	0,859
H83	Other diseases of inner ear	-0,165	-0,369	0,040	0,110	0,848
T81	Complications of procedures, not elsewhere classified	-0,181	-0,350	-0,011	0,040	0,834
I20	Angina pectoris	-0,189	-0,316	-0,061	0,000	0,828
R19	Other symptoms and signs involving the digestive system and abdomen	-0,226	-0,477	0,025	0,080	0,798
M16	Coxarthrosis [arthrosis of hip]	-0,229	-0,383	-0,076	0,000	0,795
S46	Injury of blood vessels at shoulder and upper arm level	-0,278	-0,534	-0,023	0,030	0,757
S43	Dislocation and sprain of joints and ligaments of shoulder girdle	-0,283	-0,582	0,016	0,060	0,754
E66	Obesity	-0,287	-0,496	-0,079	0,010	0,751
M50	Cervical disc disorders	-0,290	-0,657	0,076	0,120	0,748
T84	Complications of internal orthopedic prosthetic devices, implants and grafts	-0,355	-0,593	-0,116	0,000	0,701
T88	Other complications of surgical and medical care, not elsewhere classified	-0,359	-0,635	-0,082	0,010	0,698
E04	Other nontoxic goiter	-0,367	-0,774	0,040	0,080	0,693
R32	Unspecified urinary incontinence	-0,496	-0,985	-0,007	0,050	0,609
L50	Urticaria	-0,531	-1,192	0,129	0,120	0,588
I46	Cardiac arrest	-0,545	-1,150	0,059	0,080	0,580
J42	Unspecified chronic bronchitis	-0,566	-1,328	0,196	0,150	0,568
L89	Decubitus ulcer and pressure area	-0,567	-1,112	-0,022	0,040	0,567
D21	Other benign neoplasms of connective and other soft tissue	-0,660	-1,407	0,087	0,080	0,517
D68	Other coagulation defects	-0,662	-1,326	0,001	0,050	0,516
L82	Seborrhoeic keratosis	-0,690	-1,221	-0,159	0,010	0,502
F43	Reaction to severe stress, and adjustment disorders	-0,700	-1,458	0,058	0,070	0,497
N12	Tubulo-interstitial nephritis, not specified as acute or chronic	-0,701	-1,454	0,052	0,070	0,496
D69	Purpura and other haemorrhagic conditions	-0,834	-1,585	-0,083	0,030	0,434
D51	Vitamin B12 deficiency anaemia	-0,993	-1,990	0,004	0,050	0,370
J69	Pneumonitis due to solids and liquids	-1,232	-2,058	-0,406	0,000	0,292
**Medicine**						
G01	Gynecological antiinfectives and antiseptics	0,849	0,132	1,566	0,020	2,337
A09	Digestives, incl. Enzymes	0,337	-0,079	0,753	0,110	1,401

N06	Psychoanaleptics	0,227	0,132	0,322	0,000	1,255
L02	Endocrine therapy	0,226	-0,321	0,773	0,420	1,254
N07	Other nervous system drugs	0,193	0,067	0,320	0,000	1,213
A06	Drugs for constipation	0,170	0,076	0,264	0,000	1,185
A11	Vitamins	0,156	-0,108	0,421	0,250	1,169
B03	Antianemic preparations	0,150	0,054	0,246	0,000	1,162
A12	Mineral supplements	0,148	0,056	0,239	0,000	1,160
N02	Analgetics	0,139	0,033	0,245	0,010	1,149
N04	Anti-parkinson drugs	0,139	-0,031	0,309	0,110	1,149
B01	Antithrombotic agents	0,097	0,008	0,187	0,030	1,102
N05	Psycholeptics	0,056	-0,031	0,144	0,210	1,058
C08	Calcium channel blockers	0,051	-0,028	0,131	0,210	1,052
H02	Corticosteorids for systemic use	-0,092	-0,180	-0,003	0,040	0,912
S02	Otologicals	-0,096	-0,215	0,023	0,110	0,908
M01	Antiinflammatory and antitheumatic products	-0,114	-0,202	-0,025	0,010	0,892
C05	Vasoprotectives	-0,138	-0,234	-0,041	0,010	0,871
M02	Topical products for joint and muscular pain	-0,192	-0,373	-0,011	0,040	0,825
P02	Anthelmintics	-0,566	-0,989	-0,142	0,010	0,568
	Baseline risk (constant term)	-5,652	-5,780	-5,525	0,000	

Table S4 Identified predictors for hip Fracture risk (A-D)

A: Females Aged 45-64 years

Risk factor	Name	Coefficient	Confidence interval		p-value	OR
**ICD-10/ATC code**			**Lower**	**Upper**		
**Age**	45-49	Ref	-	-	-	1
	50-54	1,434	0,548	2,321	0,000	4,195
	55-59	1,960	1,109	2,811	0,000	7,099
	60-64	2,334	1,490	3,178	0,000	10,319
**Diagnosis**						
H19	Disorders of sclera and cornea in diseases classified elsewhere	2,039	0,595	3,483	0,010	7,683
H46	Optic neuritis	1,844	0,475	3,214	0,010	6,322
C54	Malignant neoplasm of corpus uteri	1,782	0,751	2,813	0,000	5,942
S03	Dislocation and sprain of joints and ligaments of head	1,778	0,720	2,835	0,000	5,918
D33	Benign neoplasm of brain and other parts of central nervous system	1,773	0,600	2,946	0,000	5,888
M95	Other acquired deformities of musculoskeletal system and connective tissue	1,720	0,515	2,926	0,010	5,585
J93	Pneumothorax	1,602	0,371	2,834	0,010	4,963
K02	Dental caries	1,540	0,641	2,439	0,000	4,665
I45	Other conduction disorders	1,362	-0,067	2,790	0,060	3,904
B37	Candidiasis	1,317	0,401	2,233	0,000	3,732
S72	Fracture of femur	1,266	0,440	2,092	0,000	3,547
N10	Acute pyelonephritis	1,201	0,317	2,085	0,010	3,323
G40	Epilepsy	1,198	0,588	1,807	0,000	3,313
K26	Duodenal ulcer	1,173	0,033	2,314	0,040	3,232
S32	Fracture of lumbar spine and pelvis	1,172	0,365	1,980	0,000	3,228
E83	Disorders of mineral metabolism	1,071	0,092	2,051	0,030	2,918
S42	Fracture of shoulder and upper arm	1,000	0,466	1,534	0,000	2,718
L97	Ulcer of lower limb, not elsewhere classified	0,956	-0,243	2,155	0,120	2,601
E16	Other disorders of pancreatic internal secretion	0,905	-0,245	2,056	0,120	2,472
G35	Multiple sclerosis	0,902	-0,087	1,891	0,070	2,465
K76	Other diseases of liver	0,818	0,025	1,610	0,040	2,266
H26	Other cataract	0,761	-0,044	1,566	0,060	2,140

R94	Abnormal results of function studies	0,756	-0,350	1,862	0,180	2,130
S92	Fracture of foot and toe, except ankle	0,721	0,241	1,200	0,000	2,056
G45	Transient cerebral ischemic attacks and related syndromes	0,711	-0,129	1,552	0,100	2,036
S52	Fracture of forearm	0,710	0,257	1,164	0,000	2,034
L60	Nail disorders	0,702	-0,551	1,954	0,270	2,018
R91	Abnormal findings on diagnostic imaging of lung	0,563	-0,142	1,268	0,120	1,756
K70	Alcoholic liver disease	0,543	-0,507	1,593	0,310	1,721
R60	Edema, not elsewhere classified	0,503	-0,501	1,507	0,330	1,654
S01	Open wound of head	0,475	-0,038	0,989	0,070	1,608
D12	Benign neoplasm of colon, rectum, anus and anal canal	0,412	-0,125	0,950	0,130	1,510
I95	Hypotension	0,405	-0,966	1,776	0,560	1,499
R18	Ascites	0,344	-1,011	1,698	0,620	1,411
J44	Other chronic obstructive pulmonary disease	0,243	-0,402	0,888	0,460	1,275
F10	Mental and behavioural disorders due to use of alcohol	0,205	-0,471	0,881	0,550	1,228
E87	Other disorders of fluid, electrolyte and acid-base balance	0,071	-0,668	0,810	0,850	1,074
**Medicine**						
A09	Digestives, incl. Enzymes	1,345	0,339	2,350	0,010	3,838
M04	Antigout preparations	0,727	-0,116	1,570	0,090	2,069
N07	Other nervous system drugs	0,641	0,254	1,028	0,000	1,898
A12	Mineral supplements	0,447	0,047	0,847	0,030	1,564
N05	Psycholeptics	0,312	-0,019	0,644	0,070	1,366
G03	Sex hormones and modulators of the genital system	-0,700	-1,021	-0,379	0,000	0,497
B02	Antihemorrhagics	-1,163	-2,067	-0,258	0,010	0,313
	Baseline risk (constant term)	-10,051	-10,883	-9,219	0,000	

B: Males Aged 45-64 years

Risk factor	Name	Coefficient	Confidence interval		p-value	OR
**ICD-10/ATC code**			**Lower**	**Upper**		
**Age**	45-49	Ref	-	-	-	1
	50-54	0,720	0,191	1,249	0,010	2,054
	55-59	0,845	0,334	1,355	0,000	2,328
	60-64	1,250	0,751	1,750	0,000	3,490
**Diagnosis**						
E16	Other disorders of pancreatic internal secretion	1,657	0,748	2,566	0,000	5,244
H47	Other disorders of optic [2nd] nerve and visual pathways	1,644	0,569	2,719	0,000	5,176
J11	Influenza, virus not identified	1,582	0,418	2,745	0,010	4,865
S72	Fracture of femur	1,552	0,961	2,143	0,000	4,721
Q21	Congenital malformations of cardiac septa	1,480	0,261	2,699	0,020	4,393
B23	Human immunodeficiency virus [HIV] disease resulting in other conditions	1,455	0,415	2,495	0,010	4,284
J22	Unspecified acute lower respiratory infection	1,449	-0,014	2,912	0,050	4,259
J01	Acute sinusitis	1,414	0,396	2,433	0,010	4,112
G35	Multiple sclerosis	1,328	0,285	2,371	0,010	3,773
D14	Benign neoplasm of middle ear and respiratory system	1,308	0,288	2,328	0,010	3,699
M21	Other acquired deformities of limbs	1,156	0,391	1,922	0,000	3,177
K22	Other diseases of oesophagus	1,132	0,376	1,887	0,000	3,102
G95	Other diseases of spinal cord	1,086	-0,548	2,721	0,190	2,962
M05	Seropositive rheumatoid arthritis	1,052	-0,055	2,160	0,060	2,863
D69	Purpura and other haemorrhagic conditions	0,997	-0,244	2,238	0,120	2,710
C18	Malignant neoplasm of colon	0,991	-0,270	2,251	0,120	2,694
H49	Paralytic strabismus	0,976	-0,477	2,430	0,190	2,654
J93	Pneumothorax	0,967	0,009	1,926	0,050	2,630
F10	Mental and behavioural disorders due to use of alcohol	0,925	0,484	1,367	0,000	2,522
R18	Ascites	0,910	-0,119	1,939	0,080	2,484
I95	Hypotension	0,892	-0,062	1,846	0,070	2,440
H83	Other diseases of inner ear	0,881	-0,017	1,780	0,050	2,413
H40	Glaucoma	0,871	-0,106	1,848	0,080	2,389
M66	Spontaneous rupture of synovium and tendon	0,868	-0,458	2,194	0,200	2,382
M13	Other arthritis	0,864	0,087	1,641	0,030	2,373

I82	Other venous embolism and thrombosis	0,772	-0,316	1,860	0,160	2,164
S42	Fracture of shoulder and upper arm	0,764	0,302	1,225	0,000	2,147
F17	Mental and behavioural disorders due to use of tobacco	0,688	0,121	1,256	0,020	1,990
I60	Subarachnoid haemorrhage	0,669	-0,643	1,981	0,320	1,952
C34	Malignant neoplasm of bronchus and lung	0,611	-0,939	2,161	0,440	1,842
H26	Other cataract	0,577	-0,216	1,370	0,150	1,781
F33	Recurrent depressive disorder	0,568	-0,354	1,491	0,230	1,765
H35	Other retinal disorders	0,561	-0,309	1,431	0,210	1,752
R41	Other symptoms and signs involving cognitive functions and awareness	0,545	-0,318	1,407	0,220	1,725
G82	Paraplegi and tetraplegi	0,535	-0,835	1,906	0,440	1,707
S82	Fracture of lower leg, including ankle	0,535	0,038	1,032	0,030	1,707
L97	Ulcer of lower limb, not elsewhere classified	0,530	-0,402	1,461	0,270	1,699
I63	Cerebral infarction	0,488	-0,161	1,137	0,140	1,629
S02	Fracture of skull and facial bones	0,478	-0,183	1,139	0,160	1,613
D50	Iron deficiency anaemia	0,468	-0,535	1,471	0,360	1,597
S52	Fracture of forearm	0,448	-0,079	0,975	0,100	1,565
K52	Other noninfective gastroenteritis and colitis	0,434	-0,352	1,220	0,280	1,543
E10	Type 1 diabetes mellitus	0,428	-0,389	1,244	0,300	1,534
S20	Superficial injury of thorax	0,426	-0,073	0,925	0,090	1,531
N17	Acute renal failure	0,375	-0,662	1,412	0,480	1,455
G40	Epilepsy	0,355	-0,304	1,013	0,290	1,426
R29	Other symptoms and signs involving the nervous and musculoskeletal systems	0,344	-0,154	0,842	0,180	1,411
J15	Bacterial pneumonia, not elsewhere classified	0,330	-0,383	1,044	0,360	1,391
J90	Pleural effusion, not elsewhere classified	0,318	-0,973	1,609	0,630	1,374
I48	Atrial fibrillation and flutter	0,315	-0,251	0,881	0,280	1,370
T93	Sequelae of injuries of lower limb	0,311	-0,256	0,877	0,280	1,365
G62	Other polyneuropathies	0,299	-0,471	1,069	0,450	1,349
I69	Sequelae of cerebrovascular disease	0,257	-0,436	0,950	0,470	1,293
H36	Retinal disorders in diseases classified elsewhere	0,234	-0,727	1,195	0,630	1,264
E66	Obesity	-1,250	-2,197	-0,303	0,010	0,287
T79	Certain early complications of trauma, not elsewhere classified	-1,307	-3,327	0,712	0,200	0,271
R33	Retention of urine	-1,396	-2,875	0,083	0,060	0,248

R73	Elevated blood glucose level	-1,396	-3,529	0,737	0,200	0,248
R51	Headache	-1,740	-3,179	-0,301	0,020	0,176
K20	Oesophagitis	-2,089	-4,286	0,108	0,060	0,124
**Medicine**						
R02	Throat preparations	0,724	0,023	1,425	0,040	2,063
N03	Antiepileptics	0,693	0,230	1,156	0,000	2,000
C02	Antihypertensives	0,524	-0,299	1,347	0,210	1,689
B03	Antianemic preparations	0,373	-0,027	0,773	0,070	1,452
A06	Drugs for constipation	0,339	-0,028	0,705	0,070	1,404
A12	Mineral supplements	0,310	-0,102	0,723	0,140	1,363
B01	Antithormbotic agents	-0,011	-0,380	0,358	0,950	0,989
C05	Vasoprotectives	-0,625	-0,997	-0,253	0,000	0,535
	Baseline risk (constant term)	-9,049	-9,503	-8,595	0,000	

C: Females Aged 65+ years

Risk factor	Name	Coefficient	Confidence interval		p-value	OR
ICD-10/ ATC code			Lower	Upper		
**Age**	65-69	Ref	-	-	-	1
	70-74	0,605	0,397	0,813	0,000	1,831
	75-79	1,075	0,877	1,272	0,000	2,930
	80+	1,998	1,812	2,183	0,000	7,374
**Diagnosis**						
C51	Malignant neoplasm of vulva	1,021	0,193	1,850	0,020	2,776
L21	Seborrheic dermatitis	0,967	0,137	1,797	0,020	2,630
H54	Blindness and low vision	0,960	0,555	1,364	0,000	2,612
J10	Influenza due to other identified influenza virus	0,928	0,274	1,583	0,010	2,529
S41	Open wound of shoulder and upper arm	0,837	0,116	1,558	0,020	2,309
J13	Pneumonia due to Streptococcus pneumoniae	0,825	0,196	1,453	0,010	2,282
G50	Disorders of trigeminal nerve	0,721	0,225	1,217	0,000	2,056
R20	Disturbances of skin sensation	0,701	0,196	1,206	0,010	2,016
S33	Dislocation and sprain of joints and ligaments of lumbar spine and pelvis	0,673	0,028	1,318	0,040	1,960
H17	Corneal scars and opacities	0,671	-0,167	1,509	0,120	1,956
T50	Poisoning by, adverse effect of and underdosing of diuretics and other and unspecified drugs, medicaments and biological substances	0,653	0,051	1,255	0,030	1,921
R18	Ascites	0,645	-0,033	1,322	0,060	1,906
G20	Parkinson disease	0,635	0,267	1,004	0,000	1,887
C79	Secondary malignant neoplasm of other and unspecified sites	0,631	-0,137	1,398	0,110	1,879
G63	Polyneuropathy in diseases classified elsewhere	0,615	0,008	1,222	0,050	1,850
D51	Vitamin B12 deficiency anaemia	0,607	0,005	1,209	0,050	1,835
M89	Other disorders of bone	0,567	0,115	1,019	0,010	1,763
F00	Dementia in Alzheimer disease	0,562	0,333	0,790	0,000	1,754
H65	Nonsuppurative otitis media	0,554	-0,057	1,166	0,080	1,740
K41	Femoral hernia	0,546	-0,128	1,221	0,110	1,726
E27	Other disorders of adrenal gland	0,545	-0,073	1,164	0,080	1,725

J90	Pleural effusion, not elsewhere classified	0,531	0,148	0,913	0,010	1,701
G91	Hydrocephalus	0,521	0,007	1,036	0,050	1,684
G30	Alzheimer disease	0,520	0,284	0,756	0,000	1,682
F17	Mental and behavioural disorders due to use of tobacco	0,517	0,173	0,861	0,000	1,677
N12	Tubulo-interstitial nephritis, not specified as acute or chronic	0,506	-0,038	1,050	0,070	1,659
D18	Hemangioma and lymphangioma, any site	0,489	-0,149	1,127	0,130	1,631
R15	Fecal incontinence	0,488	0,151	0,825	0,000	1,629
G31	Other degenerative diseases of nervous system, not elsewhere classified	0,487	-0,005	0,980	0,050	1,627
C88	Malignant immunoproliferative diseases	0,471	-0,385	1,327	0,280	1,602
R63	Symptoms and signs concerning food and fluid intake	0,461	0,178	0,745	0,000	1,586
I46	Cardiac arrest	0,451	-0,381	1,283	0,290	1,570
S32	Fracture of lumbar spine and pelvis	0,450	0,220	0,681	0,000	1,568
K70	Alcoholic liver disease	0,423	-0,199	1,046	0,180	1,527
T79	Certain early complications of trauma, not elsewhere classified	0,414	0,077	0,750	0,020	1,513
C20	Malignant neoplasm of rectum	0,403	-0,036	0,843	0,070	1,496
I51	Complications and ill-defined descriptions of heart disease	0,402	-0,002	0,807	0,050	1,495
D35	Benign neoplasm of other and unspecified endocrine glands	0,380	-0,104	0,864	0,120	1,462
S42	Fracture of shoulder and upper arm	0,372	0,217	0,526	0,000	1,451
D03	Melanoma in situ	0,361	-0,204	0,927	0,210	1,435
R74	Abnormal serum enzyme levels	0,358	-0,181	0,896	0,190	1,430
C83	Non-follicular lymphoma	0,347	-0,218	0,912	0,230	1,415
K04	Diseases of pulp and periapical tissues	0,342	-0,163	0,846	0,180	1,408
H18	Other disorders of cornea	0,341	-0,053	0,736	0,090	1,406
F10	Mental and behavioural disorders due to use of alcohol	0,328	0,021	0,635	0,040	1,388
I47	Paroxysmal tachycardia	0,323	0,038	0,608	0,030	1,381
D47	Other neoplasms of uncertain behavior of lymphoid, hematopoietic and related tissue	0,322	-0,088	0,733	0,120	1,380
J44	Other chronic obstructive pulmonary disease	0,318	0,134	0,502	0,000	1,374
R58	Hemorrhage, not elsewhere classified	0,313	-0,373	0,999	0,370	1,368
N13	Obstructive and reflux uropathy	0,311	-0,114	0,737	0,150	1,365
D30	Benign neoplasm of urinary organs	0,305	-0,200	0,810	0,240	1,357
S30	Superficial injury of abdomen, lower back, pelvis and external genitals	0,300	0,017	0,583	0,040	1,350
H34	Retinal vascular occlusions	0,299	-0,033	0,631	0,080	1,349

S81	Open wound of knee and lower leg	0,284	0,037	0,530	0,020	1,328
F01	Vascular dementia	0,269	-0,197	0,734	0,260	1,309
I25	Chronic ischemic heart disease	0,259	0,059	0,459	0,010	1,296
R04	Hemorrhage from respiratory passages	0,252	0,015	0,489	0,040	1,287
R41	Other symptoms and signs involving cognitive functions and awareness	0,246	0,037	0,455	0,020	1,279
S43	Dislocation and sprain of joints and ligaments of shoulder girdle	0,236	-0,082	0,553	0,150	1,266
F06	Other mental disorders due to brain damage and dysfunction and to physical disease	0,229	-0,200	0,659	0,300	1,257
S52	Fracture of forearm	0,226	0,098	0,354	0,000	1,254
I73	Other peripheral vascular diseases	0,222	-0,025	0,468	0,080	1,249
I27	Other pulmonary heart diseases	0,218	-0,316	0,752	0,420	1,244
G62	Other polyneuropathies	0,204	-0,124	0,532	0,220	1,226
S92	Fracture of foot and toe, except ankle	0,202	-0,030	0,433	0,090	1,224
A04	Other bacterial intestinal infections	0,201	-0,222	0,625	0,350	1,223
I34	Nonrheumatic mitral valve disorders	0,200	-0,124	0,523	0,230	1,221
S20	Superficial injury of thorax	0,190	-0,092	0,471	0,190	1,209
M54	Dorsalgia	0,181	0,010	0,352	0,040	1,198
S62	Fracture at wrist and hand level	0,178	-0,016	0,373	0,070	1,195
I61	Intracerebral haemorrhage	0,173	-0,246	0,592	0,420	1,189
F32	Depressive episode	0,168	-0,133	0,469	0,270	1,183
H91	Other and unspecified hearing loss	0,154	0,026	0,282	0,020	1,166
R53	Malaise and fatigue	0,151	-0,046	0,348	0,130	1,163
S01	Open wound of head	0,151	-0,004	0,306	0,060	1,163
S72	Fracture of femur	0,147	-0,044	0,339	0,130	1,158
A49	Bacterial infection of unspecified site	0,145	-0,097	0,388	0,240	1,156
D50	Iron deficiency anaemia	0,140	-0,133	0,413	0,310	1,150
E55	Vitamin D deficiency	0,136	-0,242	0,515	0,480	1,146
I95	Hypotension	0,133	-0,164	0,430	0,380	1,142
C44	Other and unspecified malignant neoplasm of skin	0,120	-0,066	0,306	0,210	1,127
S22	Fracture of rib(s), sternum and thoracic spine	0,120	-0,247	0,486	0,520	1,127
N18	Chronic kidney disease	0,117	-0,155	0,388	0,400	1,124
H35	Other retinal disorders	0,111	-0,037	0,259	0,140	1,117
R55	Syncope and collapse	0,108	-0,052	0,268	0,190	1,114
S70	Superficial injury of hip and thigh	0,095	-0,117	0,308	0,380	1,100
I64	Slagtilfælde uden oplysning om blødning eller infarkt	0,090	-0,171	0,352	0,500	1,094

D64	Other anaemias	0,084	-0,119	0,288	0,420	1,088
E87	Other disorders of fluid, electrolyte and acid-base balance	0,075	-0,110	0,260	0,430	1,078
E78	Disorders of lipoprotein metabolism and other lipidemias	-0,143	-0,303	0,017	0,080	0,867
R07	Pain in throat and chest	-0,158	-0,347	0,031	0,100	0,854
R06	Abnormalities of breathing	-0,166	-0,338	0,007	0,060	0,847
I20	Angina pectoris	-0,173	-0,394	0,049	0,130	0,841
S61	Open wound of wrist, hand and fingers	-0,184	-0,407	0,040	0,110	0,832
M75	Shoulder lesions	-0,194	-0,402	0,015	0,070	0,824
T84	Complications of internal orthopedic prosthetic devices, implants and grafts	-0,228	-0,501	0,046	0,100	0,796
M17	Osteoarthritis of knee	-0,241	-0,390	-0,092	0,000	0,786
E05	Thyrotoxicosis [hyperthyroidism]	-0,242	-0,533	0,050	0,100	0,785
M47	Spondylosis	-0,245	-0,532	0,042	0,090	0,783
E66	Obesity	-0,261	-0,491	-0,031	0,030	0,770
G56	Mononeuropathies of upper limb	-0,268	-0,509	-0,027	0,030	0,765
I80	Phlebitis and thrombophlebitis	-0,269	-0,568	0,031	0,080	0,764
K80	Cholelithiasis	-0,297	-0,536	-0,058	0,010	0,743
K81	Cholecystitis	-0,299	-0,827	0,229	0,270	0,742
M20	Acquired deformities of fingers and toes	-0,307	-0,585	-0,029	0,030	0,736
K58	Irritable bowel syndrome	-0,339	-0,825	0,147	0,170	0,712
N20	Calculus of kidney and ureter	-0,350	-0,776	0,076	0,110	0,705
H93	Other disorders of ear, not elsewhere classified	-0,353	-0,671	-0,035	0,030	0,703
R05	Cough	-0,369	-0,687	-0,051	0,020	0,691
L30	Andre former for dermatitis	-0,390	-0,944	0,163	0,170	0,677
G47	Sleep disorders	-0,410	-0,859	0,038	0,070	0,664
E53	Deficiency of other B group vitamins	-0,430	-1,120	0,260	0,220	0,651
N60	Benign mammary dysplasia	-0,461	-1,015	0,093	0,100	0,631
N99	Postprocedural disorders of genitourinary system, not elsewhere classified	-0,475	-1,179	0,229	0,190	0,622
M72	Fibroblastic disorders	-0,477	-0,933	-0,021	0,040	0,621
J98	Other respiratory disorders	-0,499	-1,214	0,216	0,170	0,607
L02	Cutaneous abscess, furuncle and carbuncle	-0,519	-0,990	-0,048	0,030	0,595
D62	Akut anæmi efter blødning	-0,528	-1,139	0,083	0,090	0,590
J96	Respiratory failure, not elsewhere classified	-0,532	-0,918	-0,146	0,010	0,587

T40	Poisoning by, adverse effect of and underdosing of narcotics and psychodysleptics	-0,551	-1,712	0,611	0,350	0,576
J69	Pneumonitis due to solids and liquids	-0,569	-1,750	0,611	0,340	0,566
L98	Other disorders of skin and subcutaneous tissue, not elsewhere classified	-0,576	-1,207	0,054	0,070	0,562
T13	Other injuries of lower limb, level unspecified	-0,656	-1,547	0,236	0,150	0,519
L89	Decubitus ulcer and pressure area	-0,658	-1,488	0,171	0,120	0,518
E83	Disorders of mineral metabolism	-0,671	-1,278	-0,064	0,030	0,511
S05	Injury of eye and orbit	-0,675	-1,281	-0,069	0,030	0,509
L97	Ulcer of lower limb, not elsewhere classified	-0,684	-1,294	-0,074	0,030	0,505
A08	Viral and other specified intestinal infections	-0,744	-1,748	0,261	0,150	0,475
G44	Other headache syndromes	-0,783	-1,539	-0,028	0,040	0,457
M24	Other specific joint derangements	-0,802	-1,793	0,189	0,110	0,448
M66	Spontaneous rupture of synovium and tendon	-0,842	-1,830	0,146	0,090	0,431
M16	Osteoarthritis of hip	-0,887	-1,099	-0,676	0,000	0,412
I89	Other noninfective disorders of lymphatic vessels and lymph nodes	-0,897	-1,885	0,091	0,080	0,408
N64	Other disorders of breast	-0,915	-1,662	-0,167	0,020	0,401
E89	Postprocedural endocrine and metabolic complications and disorders, not elsewhere classified	-0,916	-2,068	0,236	0,120	0,400
T39	Poisoning by, adverse effect of and underdosing of nonopioid analgesics, antipyretics and antirheumatics	-0,963	-2,121	0,195	0,100	0,382
R30	Pain associated with micturition	-0,995	-2,400	0,410	0,160	0,370
H52	Disorders of refraction and accommodation	-1,013	-1,820	-0,206	0,010	0,363
F20	Schizophrenia	-1,048	-2,454	0,358	0,140	0,351
S12	Fracture of cervical vertebra and other parts of neck	-1,074	-2,476	0,329	0,130	0,342
N31	Neuromuscular dysfunction of bladder, not elsewhere classified	-1,079	-2,501	0,342	0,140	0,340
K42	Umbilical hernia	-1,116	-2,255	0,023	0,050	0,328
M71	Other bursopathies	-1,134	-2,128	-0,140	0,030	0,322
S23	Dislocation and sprain of joints and ligaments of thorax	-1,178	-2,575	0,220	0,100	0,308
K91	Intraoperative and postprocedural complications and disorders of digestive system, not elsewhere classified	-1,203	-2,603	0,197	0,090	0,300
K65	Peritonitis	-1,225	-2,623	0,173	0,090	0,294
**Medicine**						
A05	Bile and liver therapy	1,228	0,427	2,030	0,000	3,414
A09	Digestives, incl. enzymes	0,854	0,332	1,376	0,000	2,349

H04	Pancreatic hormones	0,704	0,121	1,288	0,020	2,022
H01	Pityutart abd hypothalamic hormones and analogues	0,316	-0,046	0,678	0,090	1,372
A06	Drugs for constipation	0,247	0,137	0,358	0,000	1,280
B03	Antianemic preparations	0,208	0,095	0,320	0,000	1,231
N06	Psychoanaleptics	0,191	0,082	0,300	0,000	1,210
N02	Analgetics	0,186	0,043	0,329	0,010	1,204
B01	Antithrombotic agents	0,152	0,042	0,261	0,010	1,164
N07	Other nervous system drugs	0,128	-0,046	0,302	0,150	1,137
A12	Mineral supplements	0,116	0,011	0,220	0,030	1,123
A01	Stomatological preprations	0,111	-0,010	0,232	0,070	1,117
N05	Psycholeptics	0,096	-0,005	0,197	0,060	1,101
G03	Sex hormones and modulators of the genital system	-0,063	-0,159	0,033	0,200	0,939
H02	Corticosteorids for systemic use	-0,067	-0,175	0,040	0,220	0,935
M01	Antiinflammatory and antirheumatic products	-0,078	-0,185	0,028	0,150	0,925
C05	Vasoprotectives	-0,084	-0,194	0,025	0,130	0,919
S03	Ophthalmological and otological preparations	-0,088	-0,236	0,061	0,250	0,916
R01	Nasal preparations	-0,097	-0,209	0,014	0,090	0,908
D06	Antibiotics and chemotherapeutics for dermatological use	-0,101	-0,206	0,003	0,060	0,904
J05	Antivirals for systemic use	-0,153	-0,285	-0,020	0,020	0,858
C10	Lipid modifying agents	-0,161	-0,263	-0,059	0,000	0,851
C01	Cardiac therapy	-0,199	-0,339	-0,059	0,010	0,820
J01	Antiinfectives for systemic use	-0,216	-0,392	-0,040	0,020	0,806
R02	Throat preparations	-0,243	-0,553	0,067	0,120	0,784
	Baseline risk (constant term)	-6,250	-6,491	-6,009	0,000	

D: Males Aged 65+ years

Risk factor	Name	Coefficient	Cofidence interval		p-value	OR
**ICD-10/ATC-code**			**Lower**	**Upper**		
**Age**	65-69	Ref	-	-	-	1
	70-74	0,439	0,208	0,669	0,000	1,551
	75-79	1,015	0,800	1,230	0,000	2,759
	80+	1,757	1,552	1,962	0,000	5,795
**Diagnosis**						
D46	Myelodysplastic syndromes	1,352	0,669	2,036	0,000	3,865
T30	Burn and corrosion, body region unspecified	1,347	0,345	2,348	0,010	3,846
D29	Benign neoplasm of male genital organs	1,059	0,126	1,992	0,030	2,883
L73	Other follicular disorders	0,961	-0,041	1,962	0,060	2,614
C88	Malignant immunoproliferative diseases	0,946	0,230	1,663	0,010	2,575
B00	Herpesviral [herpes simplex] infections	0,867	-0,050	1,785	0,060	2,380
N99	Postprocedural disorders of genitourinary system, not elsewhere classified	0,788	0,201	1,375	0,010	2,199
M24	Other specific joint derangements	0,732	0,124	1,340	0,020	2,079
S72	Fracture of femur	0,731	0,484	0,978	0,000	2,077
I61	Intracerebral haemorrhage	0,728	0,381	1,076	0,000	2,071
J37	Chronic laryngitis and laryngotracheitis	0,728	-0,053	1,508	0,070	2,071
M05	Seropositive rheumatoid arthritis	0,692	0,149	1,236	0,010	1,998
J14	Pneumonia due to Haemophilus influenzae	0,691	-0,141	1,524	0,100	1,996
G95	Other diseases of spinal cord	0,651	-0,182	1,483	0,130	1,917
R74	Abnormal serum enzyme levels	0,606	0,065	1,147	0,030	1,833
C67	Malignant neoplasm of bladder	0,600	0,200	1,001	0,000	1,822
L28	Lichen simplex chronicus and prurigo	0,598	-0,339	1,535	0,210	1,818
M31	Other necrotizing vasculopathies	0,576	-0,060	1,213	0,080	1,779
D13	Benign neoplasm of other and ill-defined parts of digestive system	0,575	0,048	1,102	0,030	1,777
G91	Hydrocephalus	0,574	0,086	1,062	0,020	1,775
G60	Hereditary and idiopathic neuropathy	0,573	-0,449	1,595	0,270	1,774
E16	Other disorders of pancreatic internal secretion	0,566	0,082	1,049	0,020	1,761
H52	Disorders of refraction and accommodation	0,561	0,061	1,062	0,030	1,752

S42	Fracture of shoulder and upper arm	0,545	0,301	0,789	0,000	1,725
T79	Certain early complications of trauma, not elsewhere classified	0,540	0,170	0,910	0,000	1,716
N18	Chronic kidney disease	0,537	0,311	0,762	0,000	1,711
M21	Other acquired deformities of limbs	0,532	0,080	0,985	0,020	1,702
T17	Foreign body in respiratory tract	0,521	-0,263	1,305	0,190	1,684
R63	Symptoms and signs concerning food and fluid intake	0,498	0,166	0,830	0,000	1,645
N21	Calculus of lower urinary tract	0,481	0,072	0,890	0,020	1,618
F20	Schizophrenia	0,477	-0,380	1,334	0,280	1,611
B02	Zoster	0,476	-0,113	1,065	0,110	1,610
T18	Foreign body in alimentary tract	0,463	-0,037	0,964	0,070	1,589
S32	Fracture of lumbar spine and pelvis	0,454	0,112	0,796	0,010	1,575
M06	Other rheumatoid arthritis	0,453	-0,078	0,983	0,090	1,573
G20	Parkinson disease	0,446	0,046	0,847	0,030	1,562
F00	Dementia in Alzheimer disease	0,429	0,079	0,780	0,020	1,536
J93	Pneumothorax	0,422	-0,084	0,928	0,100	1,525
I63	Cerebral infarction	0,420	0,246	0,594	0,000	1,522
M86	Osteomyelitis	0,419	-0,277	1,115	0,240	1,520
G31	Other degenerative diseases of nervous system, not elsewhere classified	0,394	-0,100	0,887	0,120	1,483
S52	Fracture of forearm	0,392	0,129	0,655	0,000	1,480
D14	Benign neoplasm of middle ear and respiratory system	0,385	-0,146	0,915	0,160	1,470
J22	Unspecified acute lower respiratory infection	0,380	-0,244	1,004	0,230	1,462
K85	Acute pancreatitis	0,377	-0,048	0,801	0,080	1,458
I15	Secondary hypertension	0,368	-0,110	0,847	0,130	1,445
D23	Other benign neoplasms of skin	0,367	0,004	0,730	0,050	1,443
K02	Dental caries	0,365	-0,246	0,976	0,240	1,441
F17	Mental and behavioural disorders due to use of tobacco	0,352	0,001	0,703	0,050	1,422
S82	Fracture of lower leg, including ankle	0,342	0,055	0,629	0,020	1,408
K74	Fibrosis and cirrhosis of liver	0,340	-0,389	1,069	0,360	1,405
C34	Malignant neoplasm of bronchus and lung	0,335	-0,083	0,753	0,120	1,398
S60	Superficial injury of wrist, hand and fingers	0,327	0,068	0,586	0,010	1,387
S30	Superficial injury of abdomen, lower back, pelvis and external genitals	0,314	-0,067	0,694	0,110	1,369
S63	Dislocation and sprain of joints and ligaments at wrist and hand level	0,304	-0,015	0,622	0,060	1,355
J44	Other chronic obstructive pulmonary disease	0,296	0,105	0,488	0,000	1,344

F41	Other anxiety disorders	0,294	-0,305	0,893	0,340	1,342
D50	Iron deficiency anaemia	0,292	-0,028	0,613	0,070	1,339
K43	Ventral hernia	0,292	-0,074	0,657	0,120	1,339
R18	Ascites	0,288	-0,488	1,064	0,470	1,334
J91	Pleural effusion in conditions classified elsewhere	0,276	-0,476	1,028	0,470	1,318
T07	Unspecified multiple injuries	0,272	-0,271	0,814	0,330	1,313
K04	Diseases of pulp and periapical tissues	0,253	-0,202	0,708	0,280	1,288
K26	Duodenal ulcer	0,251	-0,168	0,670	0,240	1,285
F10	Mental and behavioural disorders due to use of alcohol	0,244	-0,013	0,502	0,060	1,276
J90	Pleural effusion, not elsewhere classified	0,242	-0,145	0,630	0,220	1,274
G62	Other polyneuropathies	0,241	-0,049	0,530	0,100	1,273
L97	Ulcer of lower limb, not elsewhere classified	0,240	-0,157	0,637	0,240	1,271
G30	Alzheimer disease	0,239	-0,139	0,617	0,220	1,270
I95	Hypotension	0,232	-0,088	0,551	0,160	1,261
R53	Malaise and fatigue	0,220	-0,006	0,447	0,060	1,246
R41	Other symptoms and signs involving cognitive functions and awareness	0,210	-0,059	0,478	0,130	1,234
E55	Vitamin D deficiency	0,183	-0,310	0,676	0,470	1,201
S22	Fracture of rib(s), sternum and thoracic spine	0,181	-0,141	0,504	0,270	1,198
D64	Other anaemias	0,166	-0,058	0,389	0,150	1,181
S20	Superficial injury of thorax	0,161	-0,133	0,456	0,280	1,175
S01	Open wound of head	0,149	-0,030	0,327	0,100	1,161
I70	Atherosclerosis	0,143	-0,146	0,433	0,330	1,154
S70	Superficial injury of hip and thigh	0,140	-0,154	0,434	0,350	1,150
J18	Pneumonia, organism unspecified	0,130	-0,040	0,300	0,130	1,139
G40	Epilepsy	0,108	-0,224	0,439	0,520	1,114
E11	Type 2 diabetes mellitus	0,105	-0,068	0,279	0,230	1,111
N39	Other disorders of urinary system	0,099	-0,118	0,316	0,370	1,104
J15	Bacterial pneumonia, not elsewhere classified	0,084	-0,167	0,334	0,510	1,088
I10	Essential (primary) hypertension	0,049	-0,088	0,186	0,480	1,050
R29	Other symptoms and signs involving the nervous and musculoskeletal systems	0,005	-0,186	0,197	0,960	1,005
R67	Findings in the assessment of general functional ability	-0,054	-0,255	0,147	0,600	0,947
D12	Benign neoplasm of colon, rectum, anus and anal canal	-0,178	-0,353	-0,002	0,050	0,837
I20	Angina pectoris	-0,223	-0,418	-0,027	0,030	0,800
R05	Cough	-0,271	-0,648	0,106	0,160	0,763

M75	Shoulder lesions	-0,274	-0,516	-0,032	0,030	0,760
A41	Other sepsis	-0,324	-0,574	-0,073	0,010	0,723
R07	Pain in throat and chest	-0,328	-0,557	-0,098	0,010	0,720
M65	Synovitis and tenosynovitis	-0,359	-0,787	0,069	0,100	0,698
G56	Mononeuropathies of upper limb	-0,371	-0,705	-0,037	0,030	0,690
M72	Fibroblastic disorders	-0,379	-0,718	-0,040	0,030	0,685
T84	Complications of internal orthopedic prosthetic devices, implants and grafts	-0,443	-0,820	-0,065	0,020	0,642
S46	Injury of blood vessels at shoulder and upper arm level	-0,447	-0,901	0,007	0,050	0,640
S43	Dislocation and sprain of joints and ligaments of shoulder girdle	-0,464	-0,982	0,054	0,080	0,629
T88	Other complications of surgical and medical care, not elsewhere classified	-0,481	-0,913	-0,048	0,030	0,618
T78	Adverse effects, not elsewhere classified	-0,506	-1,138	0,125	0,120	0,603
N17	Acute renal failure	-0,512	-0,965	-0,060	0,030	0,599
E66	Obesity	-0,561	-0,915	-0,207	0,000	0,571
F06	Other mental disorders due to brain damage and dysfunction and to physical disease	-0,610	-1,316	0,097	0,090	0,543
M16	Coxarthrosis [arthrosis of hip]	-0,683	-0,955	-0,411	0,000	0,505
R32	Unspecified urinary incontinence	-0,726	-1,484	0,031	0,060	0,484
J69	Pneumonitis due to solids and liquids	-0,902	-1,908	0,104	0,080	0,406
L89	Decubitus ulcer and pressure area	-0,983	-1,890	-0,076	0,030	0,374
I67	Other cerebrovascular diseases	-1,001	-2,005	0,003	0,050	0,368
L82	Seborrhoeic keratosis	-1,028	-2,016	-0,039	0,040	0,358
D51	Vitamin B12 deficiency anaemia	-1,102	-2,509	0,305	0,120	0,332
R09	Other symptoms and signs involving the circulatory and respiratory systems	-1,108	-2,500	0,285	0,120	0,330
K11	Diseases of salivary glands	-1,171	-2,566	0,224	0,100	0,310
N08	Glomerular disorders in diseases classified elsewhere	-1,184	-2,589	0,220	0,100	0,306
D04	Carcinoma in situ of skin	-1,463	-3,431	0,505	0,150	0,232
H49	Paralytic strabismus	-1,830	-3,804	0,144	0,070	0,160
J47	Bronchiectasis	-2,035	-4,002	-0,067	0,040	0,131
**Medicine**						
N06	Psychoanaleptics	0,402	0,269	0,536	0,000	1,495
C02	Antihypertensives	0,272	0,036	0,508	0,020	1,313
N07	Other nervous system drugs	0,241	0,050	0,431	0,010	1,273
A06	Drugs for constipation	0,239	0,102	0,375	0,000	1,270

A12	Mineral supplements	0,206	0,074	0,338	0,000	1,229
N04	Anti-parkinson drugs	0,158	-0,089	0,404	0,210	1,171
A01	Stomatological preparations	0,114	-0,045	0,274	0,160	1,121
B03	Antihypertensives	0,088	-0,053	0,229	0,220	1,092
B01	Antithrombotic agents	0,073	-0,065	0,212	0,300	1,076
C08	Calcium channel blockers	0,073	-0,052	0,198	0,250	1,076
R05	Cough and cold preparations	-0,114	-0,241	0,013	0,080	0,892
M01	Antiinflammatory and antirheumatic products	-0,127	-0,253	0,000	0,050	0,881
R01	Nasal preparations	-0,149	-0,286	-0,012	0,030	0,862
S02	Otologicals	-0,154	-0,340	0,031	0,100	0,857
D11	Other dermatological preparations	-0,205	-0,493	0,083	0,160	0,815
C05	Vasoprotectives	-0,222	-0,372	-0,072	0,000	0,801
L01	Antineoplastic agents	-0,411	-0,986	0,164	0,160	0,663
	Baseline risk (constant term)	-6,880	-7,093	-6,666	0,000	

Table S5 Comparison of sensitivity and specificity between newly suggested risk cut-offs and those suggested in the original $FREM_{ver1}$ paper

	Aiming at 80% sensitivity			Aiming at 90% sensitivity			Suggested in original FREM		
	**Cut-off**	**Sensitivity***	**Specificity***	**Cut-off**	**Sensitivity***	**Specificity***	**Cut-off**	**Sensitivity***	**Specificity***
**Major osteoporosis fractures**									
Females 45-64	0.4%	81%	41%	0.3%	91%	27%	2.0%	15%	97%
Females 65-69	1.0%	84%	24%	0.9%	91%	13%	2.0%	26%	89%
Females 70-74	1.3%	80%	25%	1.2%	88%	15%	2.0%	37%	81%
Females 75-79	1.6%	79%	33%	1.4%	89%	15%	2.0%	57%	62%
Females 80+	2.5%	82%	26%	2.2%	91%	13%	2.0%	96%	7%
Males 45-64	0.2%	82%	31%	0.2%	82%	31%	2.0%	6%	99%
Males 65-69	0.4%	79%	51%	0.3%	93%	16%	2.0%	11%	98%
Males 70-74	0.5%	76%	51%	0.4%	86%	25%	2.0%	12%	97%
Males 75-79	0.6%	81%	34%	0.5%	89%	15%	2.0%	15%	95%
Males 80+	1.1%	81%	33%	1.0%	87%	25%	2.0%	46%	78%
**Hip fractures**									
Females 45-64	0.1%	31%	95%	0.1%	31%	95%	0.3%	12%	99%
Females 65-69	0.1%	84%	23%	0.1%	84%	23%	0.3%	26%	92%
Females 70-74	0.2%	80%	27%	0.1%	94%	7%	0.3%	56%	59%
Females 75-79	0.4%	76%	40%	0.2%	91%	12%	0.3%	91%	22%
Females 80+	1.1%	79%	38%	0.7%	92%	18%	0.3%	99%	3%
Males 45-64	0.1%	36%	94%	0.1%	36%	94%	0.3%	11%	99%
Males 65-69	0.1%	88%	50%	0.1%	88%	50%	0.3%	33%	93%
Males 70-74	0.1%	87%	19%	0.1%	87%	19%	0.3%	59%	83%
Males 75-79	0.3%	77%	55%	0.2%	87%	24%	0.3%	59%	60%
Males 80+	0.6%	76%	42%	0.4%	91%	18%	0.3%	94%	16%

*All analyses done in the cutoff validation cohort

Table S6 Predictive performance of FREM evaluated for four scenarios

	15-year lookback<br>1 year prediction<br>AUC (95% CI)*	5-year lookback<br>1 year prediction<br>AUC (95% CI)*	15-year lookback<br>2 year prediction<br>AUC (95% CI)*	5-year lookback<br>2 year prediction<br>AUC (95% CI)*
**Major osteoporotic fracture**				
**Females**				
45-64	0.702 (0.684; 0.719)	0.707 (0.690; 0.724)	0.691 (0.679; 0.704)	0.680 (0.668; 0.693)
65+	0.656 (0.644; 0.668)	0.647 (0.635; 0.660)	0.645 (0.636; 0.654)	0.636 (0.627; 0.645)
**Males**				
45-64	0.672 (0.645; 0.699)	0.672 (0.645; 0.699)	0.674 (0.655; 0.693)	0.651 (0.631; 0.670)
65+	0.714 (0.696; 0.731)	0.734 (0.718; 0.751)	0.721 (0.710; 0.733)	0.714 (0.702; 0.726)
**Hip fracture**				
**Females**				
45-64	0.728 (0.665; 0.790)	0.814 (0.753; 0.875)	0.794 (0.754; 0.835)	0.778 (0.738; 0.818)
65+	0.762 (0.743; 0.781)	0.761 (0.742; 0.780)	0.755 (0.741; 0.768)	0.753 (0.740; 0.766)
**Males**				
45-64	0.761 (0.693; 0.828)	0.757 (0.690; 0.824)	0.771 (0.728; 0.814)	0.753 (0.713; 0.793)
65+	0.764 (0.740; 0.787)	0.795 (0.774; 0.816)	0.769 (0.754; 0.784)	0.763 (0.748; 0.778)

* All performed on the model validation cohort

Table S7 Sensitivity and specificity of suggested risk cut-offs for four scenarios

Age groups, years	1-year prediction Suggested cut-off	15-year lookback 1-year prediction Sensitivity	15-year lookback 1-year prediction Specificity	5-year lookback 1-year prediction Sensitivity	5-year lookback 1-year prediction Specificity	2-year prediction Suggested cut-off	15-year lookback 2-year prediction Sensitivity	15-year lookback 2-year prediction Specificity	5-year lookback 2-year prediction Sensitivity	5-year lookback 2-year prediction Specificity
**Major osteoporotic fracture**										
**Females**										
45-64	0.4%	81%	41%	83%	44%	0.8%	44%	82%	29%	89%
65-69	1.0%	84%	24%	84%	20%	2.0%	21%	90%	10%	96%
70-74	1.3%	80%	25%	86%	21%	2.6%	20%	90%	12%	96%
75-79	1.6%	79%	33%	78%	35%	3.2%	21%	89%	12%	95%
80+	2.5%	82%	26%	82%	25%	5.0%	27%	84%	15%	92%
**Males**										
45-64	0.2%	84%	31%	70%	52%	0.4%	41%	85%	25%	94%
65-69	0.4%	79%	51%	66%	57%	0.8%	29%	90%	20%	95%
70-74	0.5%	76%	51%	64%	57%	1.0%	31%	89%	21%	94%
75-79	0.6%	81%	34%	82%	33%	1.2%	36%	84%	29%	89%
80+	1.1%	81%	33%	84%	33%	2.2%	35%	80%	27%	86%
**Hip fracture**										
**Females**										
45-64	0.1%	31%	95%	23%	97%	0.2%	20%	98%	12%	99%
65-69	0.1%	84%	23%	96%	9%	0.2%	38%	77%	45%	72%
70-74	0.2%	80%	27%	95%	12%	0.4%	49%	80%	49%	76%
75-79	0.4%	76%	40%	84%	22%	0.8%	30%	86%	33%	85%
80+	1.1%	79%	38%	91%	21%	2.2%	37%	79%	37%	78%
**Males**										
45-64	0.1%	36%	94%	34%	97%	0.2%	29%	98%	16%	99%
65-69	0.1%	88%	50%	72%	41%	0.2%	37%	88%	29%	92%
70-74	0.1%	87%	19%	98%	9%	0.2%	69%	70%	61%	73%
75-79	0.3%	77%	55%	76%	49%	0.6%	39%	86%	33%	89%
80+	0.6%	76%	42%	86%	36%	1.2%	44%	78%	38%	81%

* All performed on the cut-off validation cohort

Supplemental figures

Figure S1: Flowchart of condition of interest for inclusion

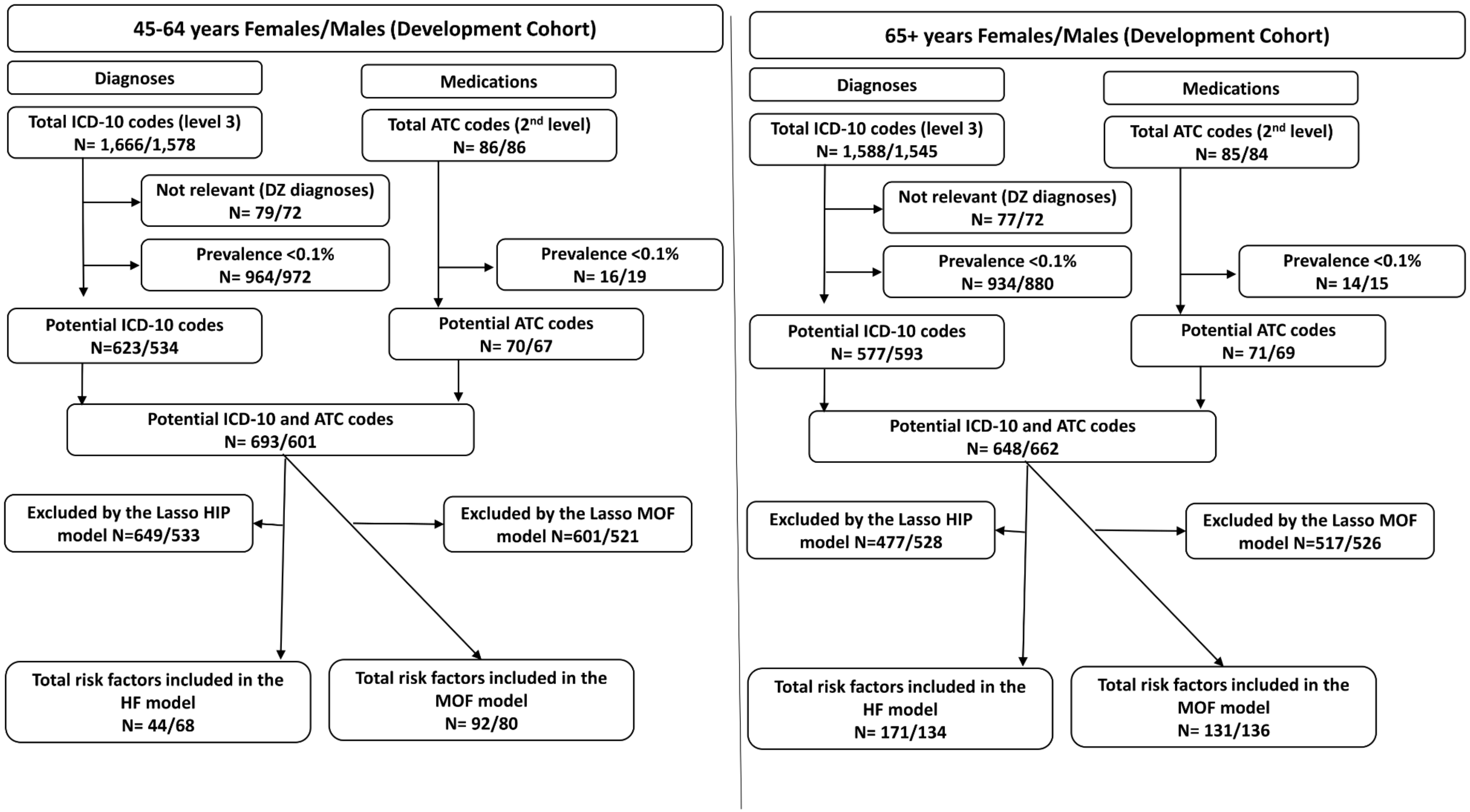

Figure S2: Comparison of area under the curve (AUC) for predicting HF between models including age only, age and diagnoses and $FREM_{ver2}$

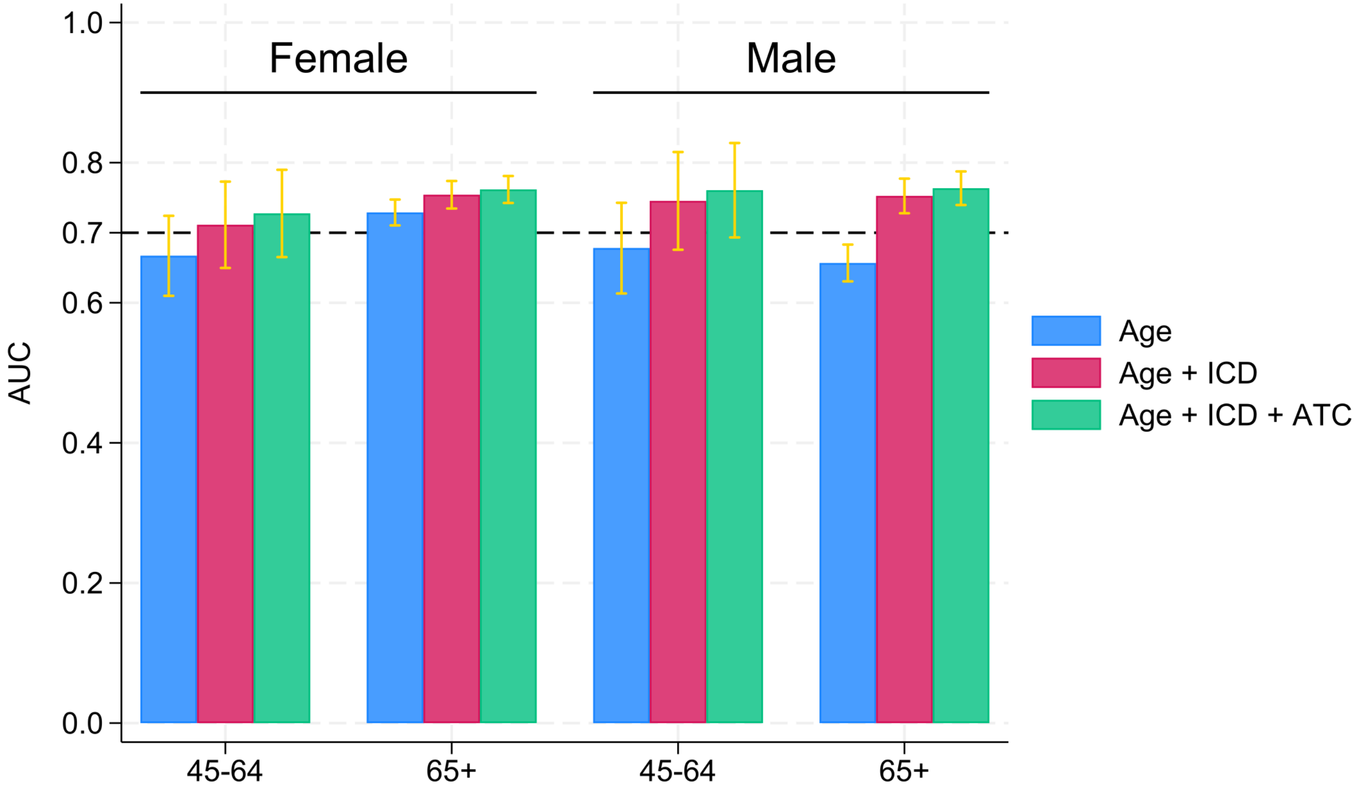

Figure S3: Positive (PPV) and inverse negative (NPV) predictive value for prediction of 1-year MOF risk evaluating cut-offs from 0.1% to 4%.

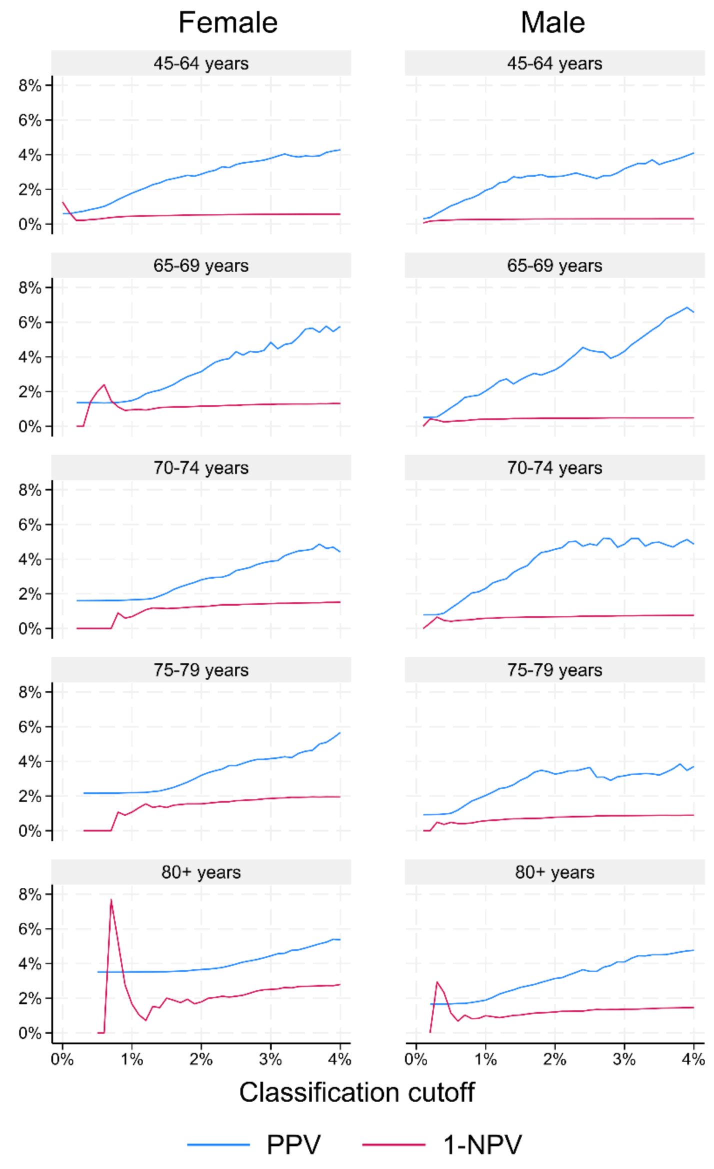

Figure S4: Positive (PPV) and inverse negative (NPV) predictive value for prediction of 1-year HF risk evaluating cut-offs from 0.1% to 4%.

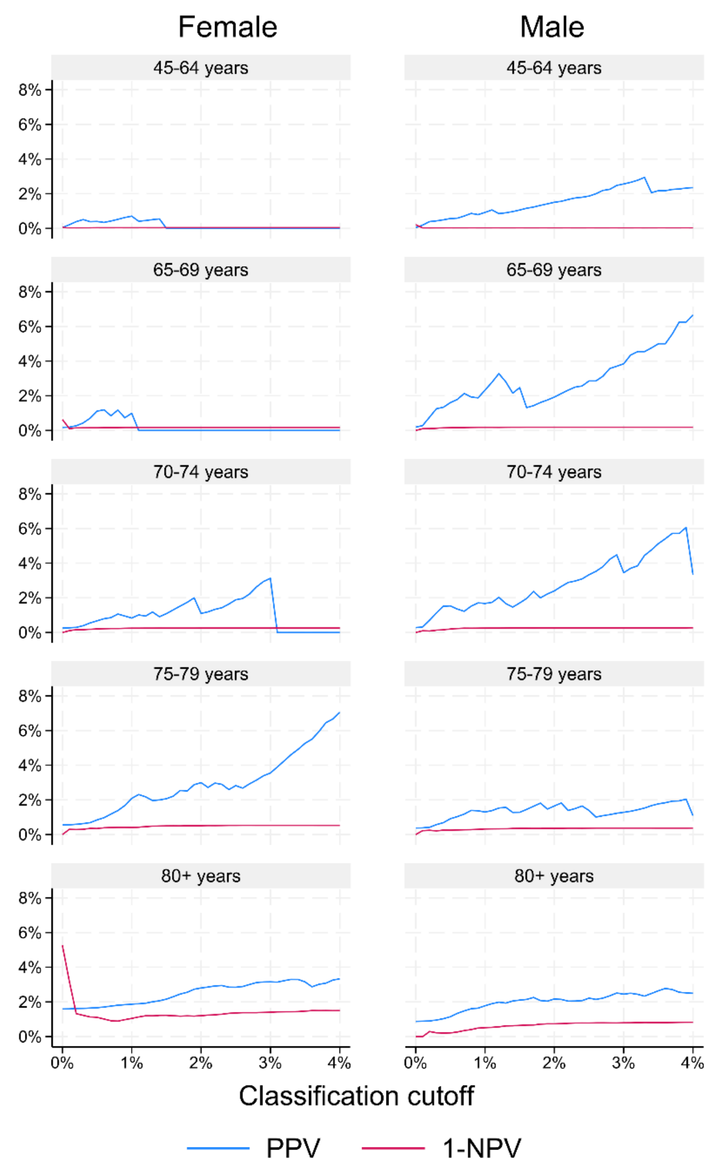

Supplement: FREM2_supplementary_final_adjustments_zjaf156 [file frem2_supplementary_final_adjustments_zjaf156.pdf]
